# Dianthi herba: a comprehensive review of its botany, traditional use, phytochemistry, and pharmacology

**DOI:** 10.1186/s13020-022-00570-2

**Published:** 2022-01-21

**Authors:** Qian Liu, Er-Huan Zang, Cong-Cong Wang, Yu-Chao Liu, Hui Niu, Yuan Gao, Min-Hui Li

**Affiliations:** 1 Department of Pharmacy, Baotou Medical College, Baotou, 014040 Inner Mongolia China; 2 Pharmaceutical Laboratory, Inner Mongolia Institute of Traditional Chinese Medicine, Hohhot, 010020 Inner Mongolia China; 3 Inner Mongolia Key Laboratory of Characteristic Geoherbs Resources Protection and Utilization, Baotou Medical College, Baotou, 014040 Inner Mongolia China; 4 Inner Mongolia Hospital of Traditional Chinese Medicine, Hohhot, 010020 Inner Mongolia China

**Keywords:** Dianthi herba, Botany, Traditional use, Phytochemistry, Pharmacology, Clinical application

## Abstract

Dianthi herba (called “*Qumai*” in Chinese) is the dried aerial part of *Dianthus superbus* L. and *Dianthus chinensis* L. The species are mainly distributed in the temperate and warm temperate regions in the northern hemisphere, and some regions in Africa and Oceania, as well as South America. However, the distribution pattern of Dianthi herba has not been reviewed. In this review, we summarize the research progress on the botany, traditional use, phytochemistry, pharmacology, toxicology, and clinical applications of Dianthi herba. Approximately 194 chemical compounds have been identified and isolated from Dianthi herba, the most important being triterpenoid saponins, flavonoids, and volatile oil compounds. These compounds possess antiviral, anticancer, antioxidant, and antimicrobial properties, inter alia. Further studies should be carried out on Dianthi herba to elucidate more of its active principles and their mechanisms of action.

**Supplementary Information:**

The online version contains supplementary material available at 10.1186/s13020-022-00570-2.

## Background

Dianthi herba is a traditional Chinese medicine (TCM) known as “*Qumai*.” According to the Pharmacopoeia of the People’s Republic of China (PRC; 2020), the authentic varieties of Dianthi herba are derived from the dried aerial parts of *Dianthus superbus* L. and *Dianthus chinensis* L., belonging to the family Caryophyllaceae, known as “*Qumai*” and “*Shizhu*” (Pharmacopoeia Commission of PRC, 2020), respectively. The first record of Dianthi herba is in *Shennong Bencaojing* (the Classic of Herbal Medicine). Traditionally, it has been used to cure diuresis, invigorate blood circulation, and regulate menstruation [1, 2]. Owing to its good efficacy, Dianthi herba is often used in combination with other TCMs to treat diseases. For example, *Gualou Qumai* pills, which contain Dianthi herba, Trichosanthis Fructus, Poria, Dioscoreae rhizoma, and Aconiti Lateralis Radix Praeparata, is a traditional prescription for treating dysuria, edema, and polydipsia [3]. Moreover, Dianthi herba is widely used in Mongolian medicine. Dianthi herba is most frequently used as a variety of “Basaga” in Mongolian medicine compound preparation [4]. Common clinical dosage forms of Dianthi herba include Shenmai-7 decoction, Clove-8 Wei powder, Zandan-4 decoction, Wu Lingzhi-5 decoction, and Digeda-4 decoction. Digeda-4 decoction has been included in the Encyclopedia of Mongolian Medicine-Mongolian Medicine and The Pharmaceutical Standards of the Ministry of Health-Mongolian Medicine Volume. The present review revealed that 194 compounds from *D. superbus* and *D. chinensis* have been reported, and they are mainly classified as saponins, flavonoids, peptides, anthraquinones, phenolic acids, amides, phenylpropanoids, and others. Modern pharmacological studies have shown that Dianthi herba has antitumor, antioxidant, antiviral, anti-inflammatory, diuretic, uterine excitatory, antimicrobial, and neuroprotective properties [5, 6].

**Fig. 1:**
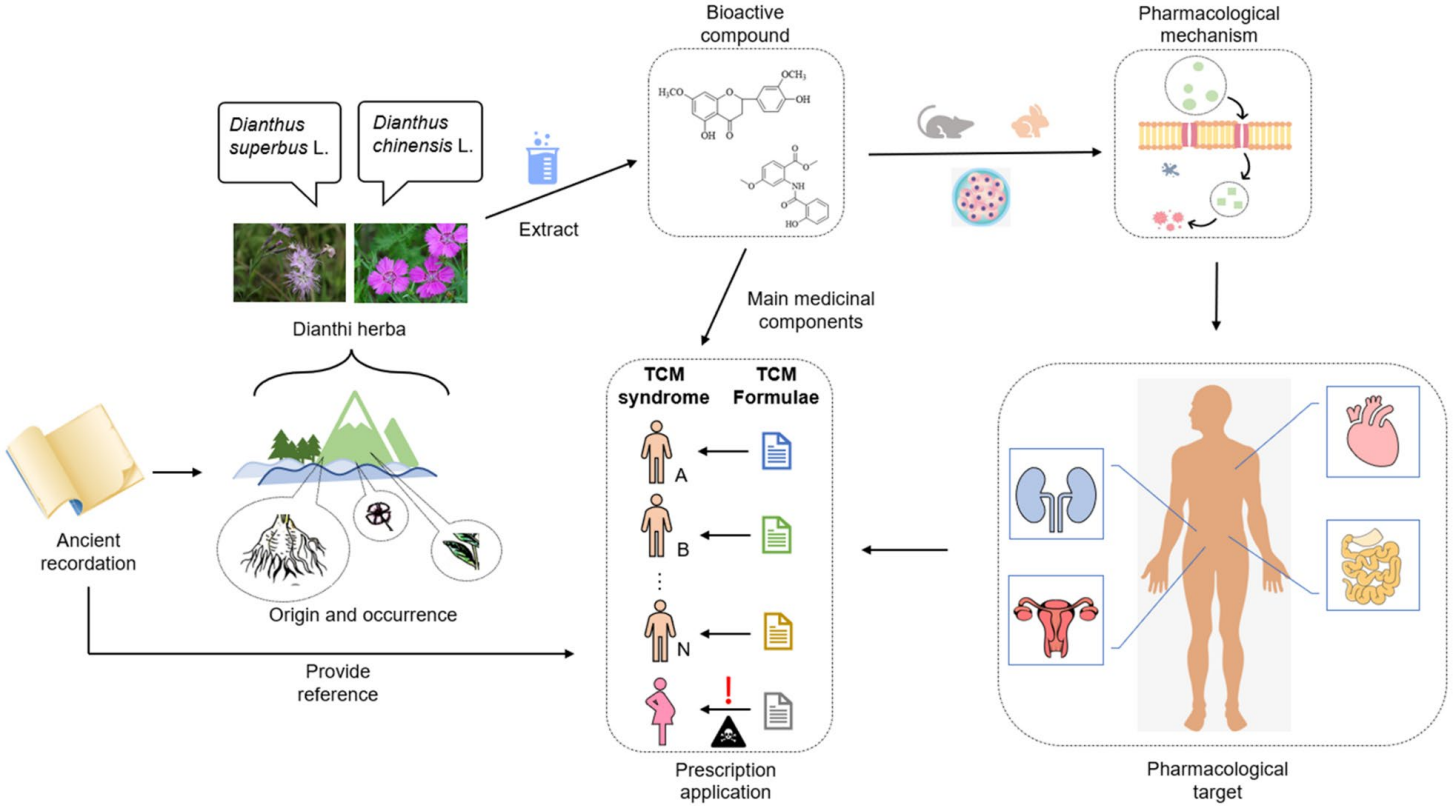
The connection of each part of the content

In this paper, we used PubMed, ScienceDirect, Web of Science, Springer, Wiley, and the China National Knowledge Infrastructure (CNKI) to collect the relevant literature on Dianthi herba from 1959 to 2021 and reviewed its botany, traditional application, phytochemistry, pharmacological properties, toxicity, and clinical applications (Fig. 1 visually shows the thinking of this paper). We aimed to provide a comprehensive review of Dianthi herba to determine its therapeutic potential and indicate directions for future research that will serve as a basis for the further development and utilization of this resource.

## Botany

**Fig. 2:**
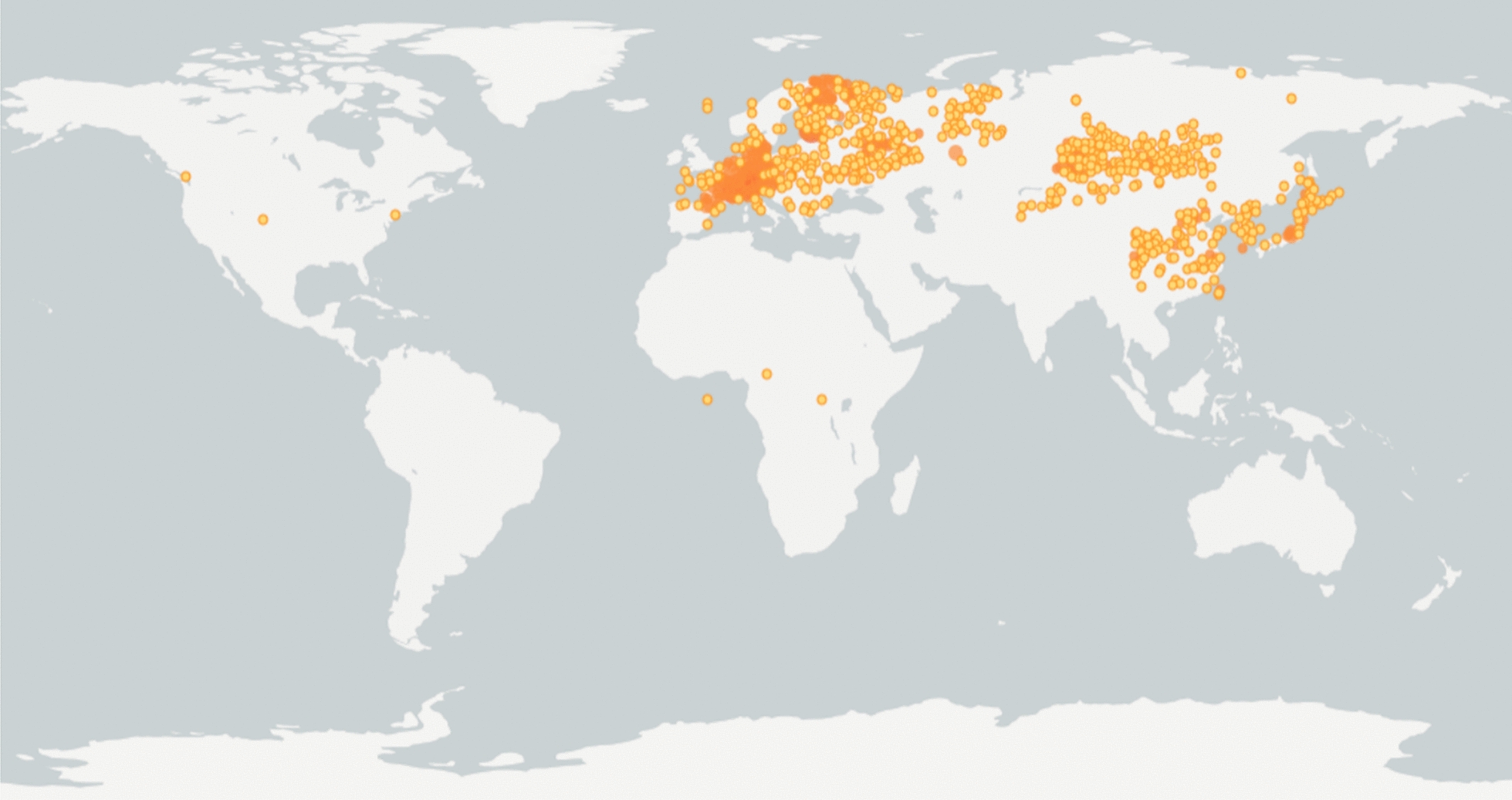
Distribution map of *D. superbus* by GBIF ( https://www.gbif.org/). The distribution proportion of *D. superbus* is 7.3% in Asia, 92.4% in Europe, and 0.3% in North America and South America. More detailed coordinate information shows this in more detail (see Additional file 1)

**Fig. 3:**
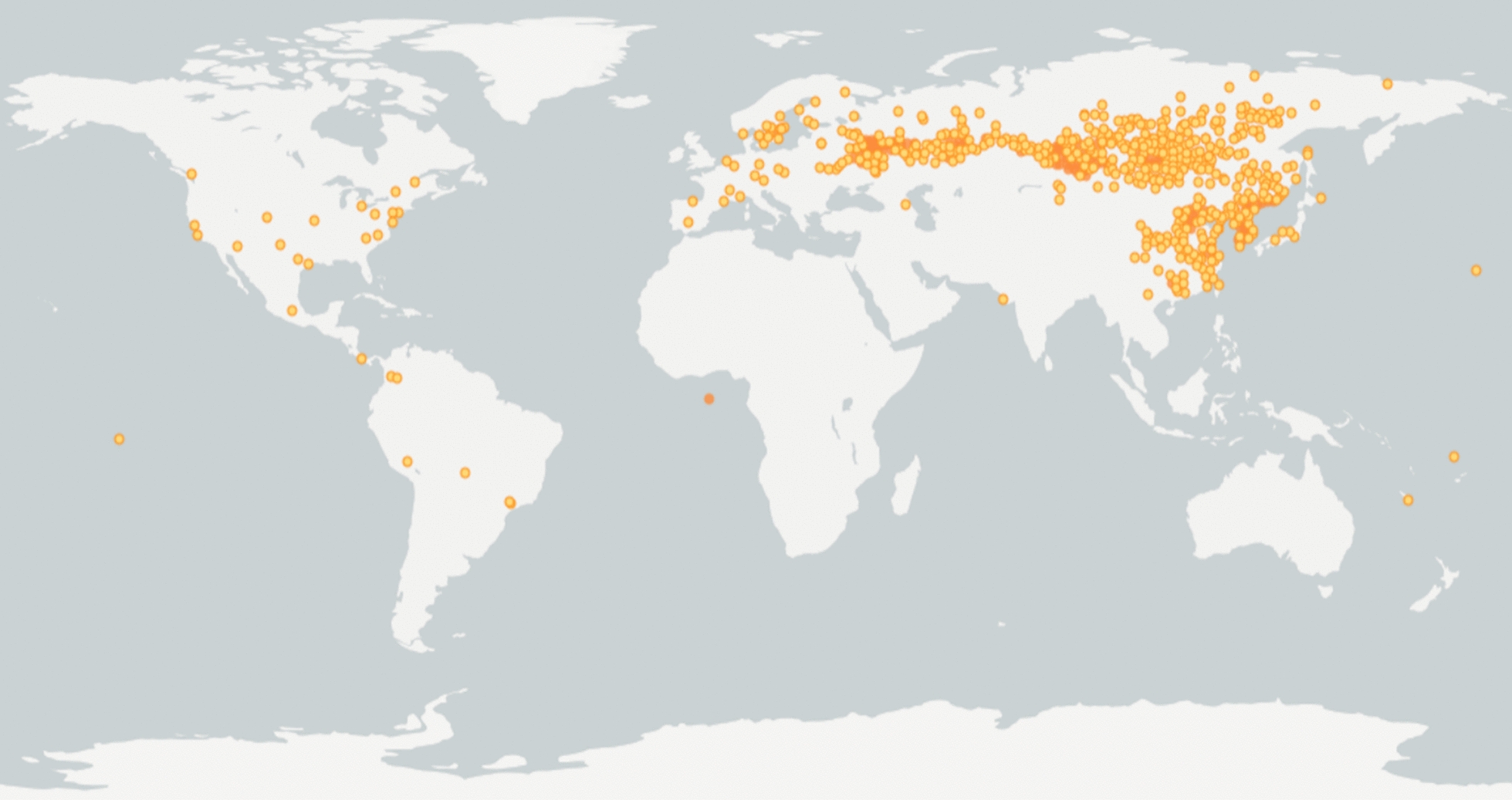
Distribution map of *D. chinensis* by GBIF ( https://www.gbif.org/). The distribution proportion of *D. chinensis* is 35.1% in Asia, 57.6% in Europe, and 4.1% in North America and 3.2% in South America. More detailed coordinate information shows this in more detail (see Additional file 1)

The family Caryophyllaceae comprises approximately 2000 plant varieties distributed worldwide. *D. superbus* is mainly distributed in the temperate and warm temperate regions of the northern hemisphere, particularly in northern Europe, central Europe, Siberia, Kazakhstan, Mongolia (western and northern), Korea, Japan, and China, and in some regions in Africa and Oceania, as well as South America (Fig. 2). *D. chinensis* inhabits regions similar to those of *D. superbus* and is mainly distributed in Kazakhstan, Korea, Mongolia, Russia, and Europe (Fig. 3). The species distribution data are from the Global Biodiversity Information Facility (GBIF)—is an international network and data infrastructure funded by the world's governments and aimed at providing anyone, anywhere, open access to data about all types of life on Earth. GBIF Secretariat currently manages and maintains GBIF.

*Dianthus superbus* is an herbaceous perennial plant, growing to a height of 50–60 cm with stems that are clumped and branched at the upper part. The leaf blades are linear-lanceolate, 5–10 × 0.3–0.5 cm in size, and the apex is acuminate and connate at the base forming a sheath. There are one to two terminal or axillary flowers with two to three pairs of bracts that are obovate and 0.6–1 × 0.4–0.5 cm in size. The apex has a long cusp, while the upper edge of the petal is lobed into a fringed shape. The plants blossom from June to September, and the fruit appears from August to October. This plant is widely cultivated in China where it grows in hilly mountain forests, forest margins, meadows, valleys, and other locations at altitudes of 400–3700 m (Flora Reipublicae Popularis Sinicae, 2019).

**Fig. 4:**
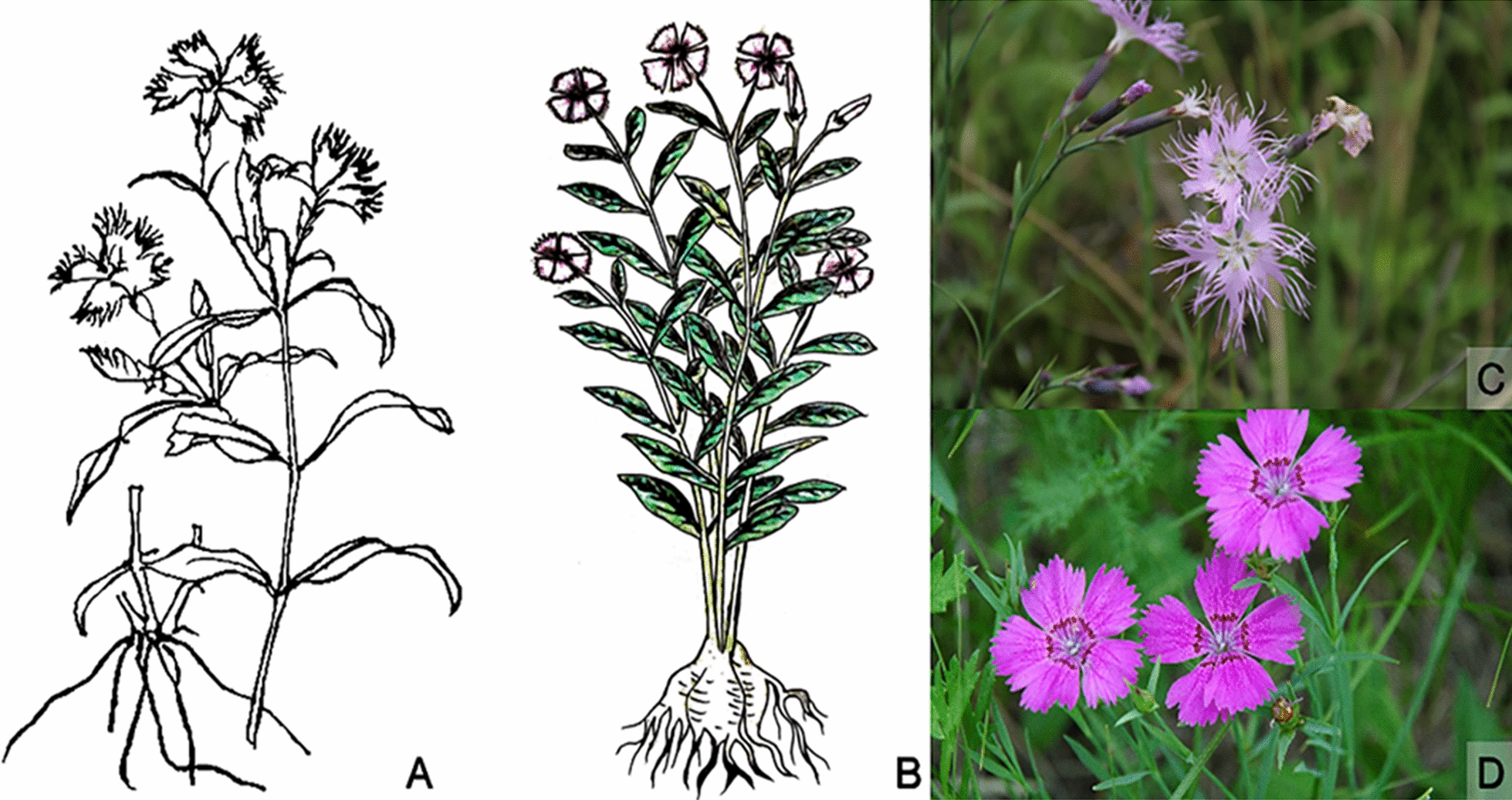
*D. superbus* recorded in *Ben Cao Pin Hui Jing Yao* (A): “*Qumai*,” also known as “*Jujumai*”,“*Daju*” and “*Yuemai*.” In ancient times, it was mainly distributed in Shandong, Shanxi, Henan, and Huaihe River Basins, with Xinjiang County and its surrounding areas in Shanxi Province as its authentic production areas; *D. chinensis* depicted in *Zhi Wen Ben Cao* (B): It was called “*Shiyangjin*” in ancient times, and was born in the wilderness. It sprouts in spring and blossoms in summer. The bud is very small and has slender flowers; The plant of *D. superbus* (C), and the plant of *D. chinensis* (D)

*Dianthus chinensis* is an herbaceous perennial plant that grows to a height of 30–50 cm, and the whole plant is hairless. The stems arise from rhizomes and are sparsely clustered with erect upper branches. The leaf blades are linear-lanceolate and 3–5 × 0.2–0.4 cm in size, with an apex that is acuminate and connate at the base forming a sheath. The flowers are solitary branchlets that are terminal or compound umbels and approximately 1–3 cm long. There are four bracts, while the length of the sepal is half, the apex has a long cusp, and the upper edge of the petal is tooth-shaped. The plant blossoms from May to June, and the fruits appear from July to September. This plant is native to northern China and currently grows in northern and southern regions, grasslands, hillside meadows, and other regions (Flora Reipublicae Popularis Sinicae, 2019). The plant morphologies of *D. superbus* and *D. chinensis* are shown in Fig. 4.

## Traditional uses

TCMs have been used to treat various diseases, and the long history of use and superior safety profile of TCM formulations have increased the confidence of public in TCMs and its acceptance. Dianthi herba is one of the few commonly used TCMs, and is recorded in ancient Chinese books and Chinese herbal medicine manuals in successive dynasties. It has been documented under different names in several well-known medicinal records, including *Shennong Bencaojing* (called “*Jujumai*”), *Guang Ya* (called “*Ziwei*”), *Ri Hua Zi Ben Cao* (called “*Shizhu*”. This is the name of “*Shizhu*” for the first time.), and *Qi Min Yao Shu* (called “*Dimian*”, because its seeds can be made into cakes to eat), and in contemporary medicinal records, such as *Zhong Hua Ben Cao* and *The Chinese Dictionary of Herbal Medicine*. *Qumai* is a combination of seeds, stems, and leaves and was first mentioned in *Shennong Bencaojing.* The textual legend of the plant is shown in *Ben Cao Jing Ji Zhu*, the plant is described to have a fruit resembling wheat. The plant morphology of a carnation “stem with distinct nodes, leaves opposite, striate, bract subcalyx, petal margins shallowly toothed” has been accurately described in the book of *Zhi Wu Ming Shi Tu Kao*. Since the Liang Dynasty, *Dianthus* has been used as a medicinal herb in the name of “*Qumai*,” which has become the main plant source of the TCM “*Qumai*.” The detailed traditional uses are shown in Table 1.

**Table 1:** The detailed traditional uses of *Dianthus superbus* L. as medicine

Species	Traditional uses	Traditional Chinese medicine with the same traditional uses	Refs
*Dianthus superbus* L	Hemorrhoid and fistula	*Nepeta cataria* L.; Lonicerae Japonicae Flos; Sophorae Flos; *Gentiana macrophylla* Pall.; Sophorae Fructus; Coptidis Rhizoma; Rehmanniae Radix; Notoginseng Radix Et Rhizoma; *Astragalus mongholicus* Bunge; Codonopsis Radix; Mume Fructus	*Ri Hua Zi Ben Cao*, 1578 C.E. [107]
	Cysts	*Astragalus mongholicus* Bunge; Codonopsis Radix; Salviae Miltiorrhizae Radix Et Rhizoma; Atractylodis Macrocephalae Rhizoma; Sparganii Rhizoma; *Curcuma phaeocaulis* Valeton; Sophorae Flavescentis Radix	[88, 108–110]
	Anti-early pregnancy	Pinelliae Rhizoma; Carthami Flos; Arnebiae Radix	[38, 111–113]
	Diabetic nephropathy	Dioscoreae Rhizoma; *Cornus officinalis* Sieb. et Zucc.; Alismatis Rhizoma; Persicae Semen	[89, 114]
	Chronic prostatitis	Angelicae Sinensis Radix; Ginseng Radix Et Rhizoma; *Epimedium brevicornu* Maxim.; *Tripterygium wilfordii* Hook. f.; Ginseng Radix Et Rhizoma Rubra	[90, 92, 115, 116]
	Pelvic inflammatory disease	Salviae Miltiorrhizae Radix Et Rhizoma; Paeoniae Radix Rubra; Violae Herba; Taraxaci Herba	[91, 117]
	Vulnerary and Amenorrhea	Angelicae Sinensis Radix; Cyperi Rhizoma; *Ligusticum chuanxiong*; Codonopsis Radix	[93, 105, 118]
	Gonorrhea	*Smilax glabra* Roxb.; Kochiae Fructus; *Portulaca oleracea* L.; *Hedyotis diffusa* Willd	[94, 106, 119]
	Antiphlogistic and Diuretic	*Phyllanthus urinaria* L.; LygodII Spora; *Emilia sonchifolia* (L.) DC	[71, 78, 95, 120]

Ancient books have described the characteristics of Dianthi herba. Dianthi herba tastes bitter, and is cold and non-toxic. Dianthi herba is mainly used as a diuretic to help relieve the pain during urination. Its clinical uses include the treatment of urinary tract infections, red and astringent urination, dysmenorrhea, red eye, eye swelling and pain, esophageal cancer, and rectal cancer. Dianthi herba has a wide range of medicinal uses and is mostly prescribed clinically (Table 2). Furthermore, Dianthi herba is used as medicine by ethnic minorities in China. Dianthi herba is recorded in *Zhonghua Materia Medica—*Mongolian Medicine Volume; it is indicated for blood fever, blood tingling, liver fever, and other diseases such as phase stroke and puerperal fever [4]. In China, Dianthi herba is sold commercially based on the external features of the dried aerial part. Currently, most medicinal products of Dianthi herba sold in the market are sourced from *D. chinensis* and *D. superbus*, but there are also products containing adulterants, such as *D. chinensis* var. *versicolor* and *D. superbus* var. *longicalycinus* [7–9].

**Table 2:** The classic prescriptions contained *D. superbus*

Preparation names	Composition crude drug names	Traditional uses	The role of Dianthi herba	The origin of ancient books
Bazheng Powder	Dianthi herba (5 g), Polygoni avicularis herba (5 g), Plantaginis semen (5 g), Gardeniae fructus (5 g), Glycyrrhizae radix et rhizoma (5 g), Rhei radix et rhizoma (5 g), talcum powder (10 g)	Treating dysuria, and stranguria ( difficulty in urination) due to hematuria	Clearing damp-heat	*Taiping Huimin Heji Jufang* (Song Dynasty, A.D. 1151)
	Dianthi herba (10 g), Plantaginis semen (10 g), Polygoni avicularis herba (10 g), Akebiae caulis*.* (10 g)	Treating cystitis, urethritis, acute prostatitis, urolithiasis and pyelonephritis	Clearing damp-heat	*TWelfare Pharmacy* (Song Dynasty, A.D. 1078–1085)
Lixiao Powder	Dianthi herba (30 g), Gardeniae fructus (15 g), Glycyrrhizae radix et rhizoma (22 g)	Clearing heat-fire, and promoting diuresis for stranguria	Alleviate diuresis	*Taiping Huimin Heji Jufang* (Song Dynasty, A.D. 1151)
*Gualouqumai* Pills	Dianthi herba (3 g), Trichosanthis Fructus (6 g), Poria (6 g), Dioscoreae rhizoma (6 g), Aconiti lateralis radix praeparata (5 g)	Treating dysuria, edema and polydipsia	Warming yang in diuresis	*Jinkui Yaolue Fanglun* (Han Dynasty, A.D. 219)
*Qumai* Decoction	Dianthi herba (30 g), Alismatis rhizoma (45 g), talcum powder (45 g), Stephaniae tetrandrae radix (23 g), Scutellariae radix (7.5 g), Rhei radix et rhizoma (7.5 g), Mantidis Oötheca (40 pieces)	Treating polydipsia, weight loss, dysuria and edema	Treating retentionofurine	*Waitai Miyao* (Tang Dynasty, A.D. 752)
	Dianthi herba (45 g), Scutellariae radix (30 g), *S. japonica* (30 g), Angelicae sinensis radix (23 g),Paeoniae radix alba (23 g), Akebiae caulis (45 g), Poria (23 g), talcum powder (23 g)	Treating stranguria due to the disturbance of dysuria	Treating retentionofurine	*Shengji Zonglu* (Song Dynasty, A.D. 1111–1117)
*Qumai* Pills	Dianthi herba (15 g), Ginseng radix et rhizoma (15 g), Rhei radix et rhizoma (15 g),Angelicae sinensis radix (15 g),Paeoniae radix alba(15 g), Cinnamomi cortex (15 g), Poria (15 g), *D. nemorosa* (23 g)	Treating peripheral edema, and congestive heart failure	Promoting blood circulation	*Jifeng Pujifang* (Song Dynasty, A.D. 960–1279)
*Qumai* Powder	Dianthi herba (30 g), Astragali radix (30 g), Asari radix et rhizoma (30 g), Paeoniae radix alba (30 g), Coicis semen (30 g), Chuanxiong rhizoma (30 g), Vignae semen (30 g)	Treatment of dysuria	Expelling abscess and dredging channels and collaterals	*Liujuan Zigui Yifang* (Qi Dynasty, A.D. 495–499)
Digeda-4 Decoction	Dianthi herba (1.25 g), *L. rotatum* (1.25 g), *N. scrophulariiflora* (1.25 g), Gardeniae fructus (1.25 g)	Cooling blood, curing sore throat, thirsty and agitated, eliminating heat in the liver and gallbladder	Clearing blood heat	*Gan Lu Si Bu* (Qi Dynasty, A.D. 1704–1788)
*Sijinqumai* Pill	Dianthi herba (10 g), Lysimachiae herba (20 g), *L. japonicum* (20 g), *Curcuma rcenyujin* (10 g), Galli gigerii endothelium corneum (5 g)	Alleviate ureteral calculi	Clearing heat and diuresis	*Lin Zhen Zhi Yan* (Dong. J.H. 1986)
Shi Wei Powder	Dianthi herba (30 g), *P. lingua* (60 g), Plantaginis semen (90 g), *M. verticillata* (60 g)	Treating gonorrhea, adverse urination, and stabbing pain when drowning	Clearing blood heat	*Zheng Zhi Hui Bu* (Qi Dynasty, A.D. 1687)

*D. nemorosa*, *Draba nemorosa* L; *S. japonica*, *Stachys japonica* Miq.; *L. rotatum*, *Lomatogonium rotatum* (L.) Fries ex Nym; *N. scrophulariiflora*, *Neopicrorhiza scrophulariiflora* (Pennell) D.Y.Hong; *L. japonicum*, *Lygodium japonicum* (Thunb.) Sw.; *P. lingua*, *Pyrrosia lingua*; *M.verticillat*, *Malva verticillata* L

## Phytochemistry

Approximately 194 compounds including saponins, flavonoids, volatile oils, and cyclic peptides have been identified in *D. superbus* and *D. chinensis*. These compounds and their corresponding structures are shown in Figs. 5, 6, 7, 8, 9, 10, 11, 12 and Table 3.

**Fig. 5:**
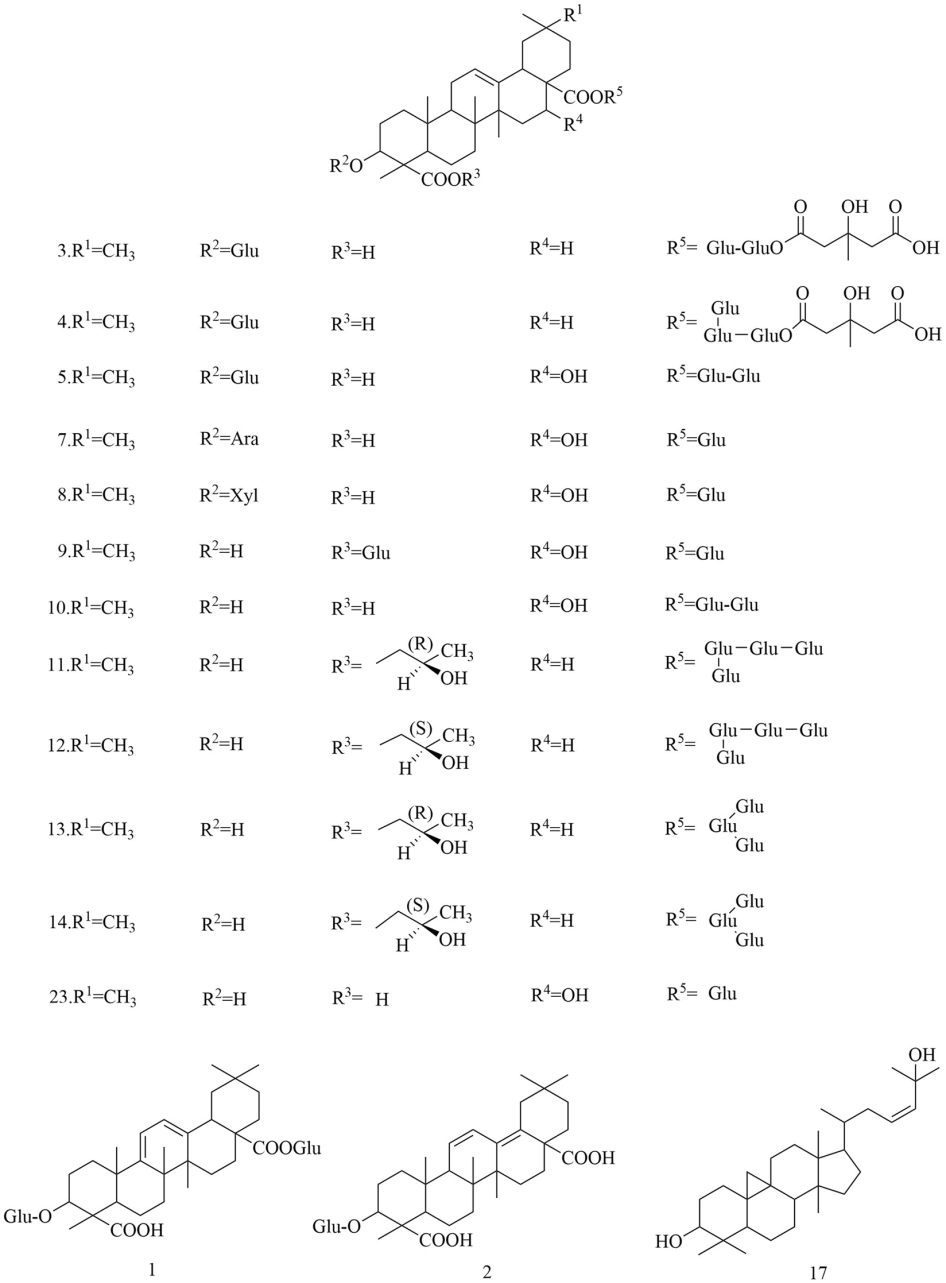

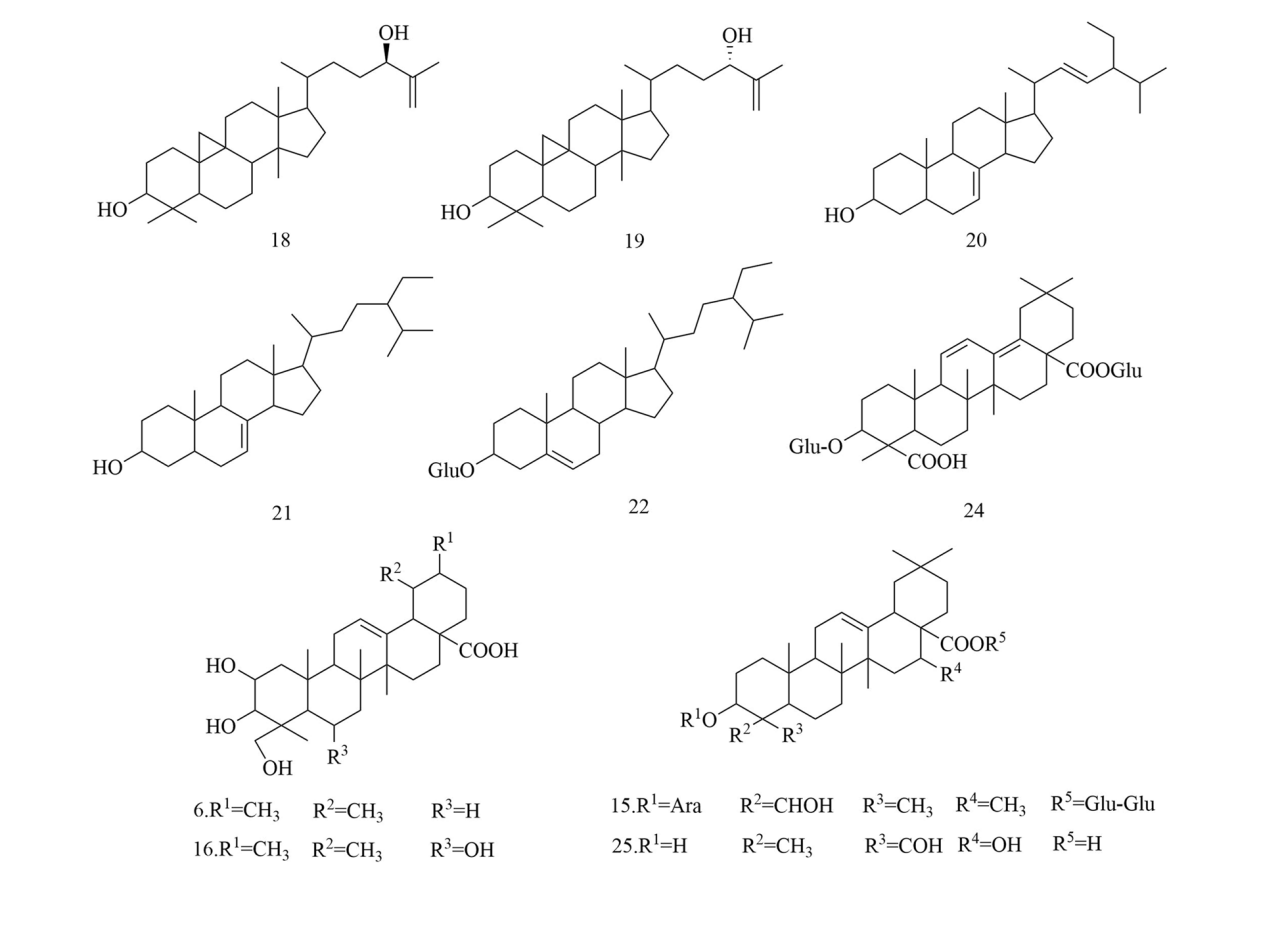
Chemical structures of saponins

**Fig. 6:**
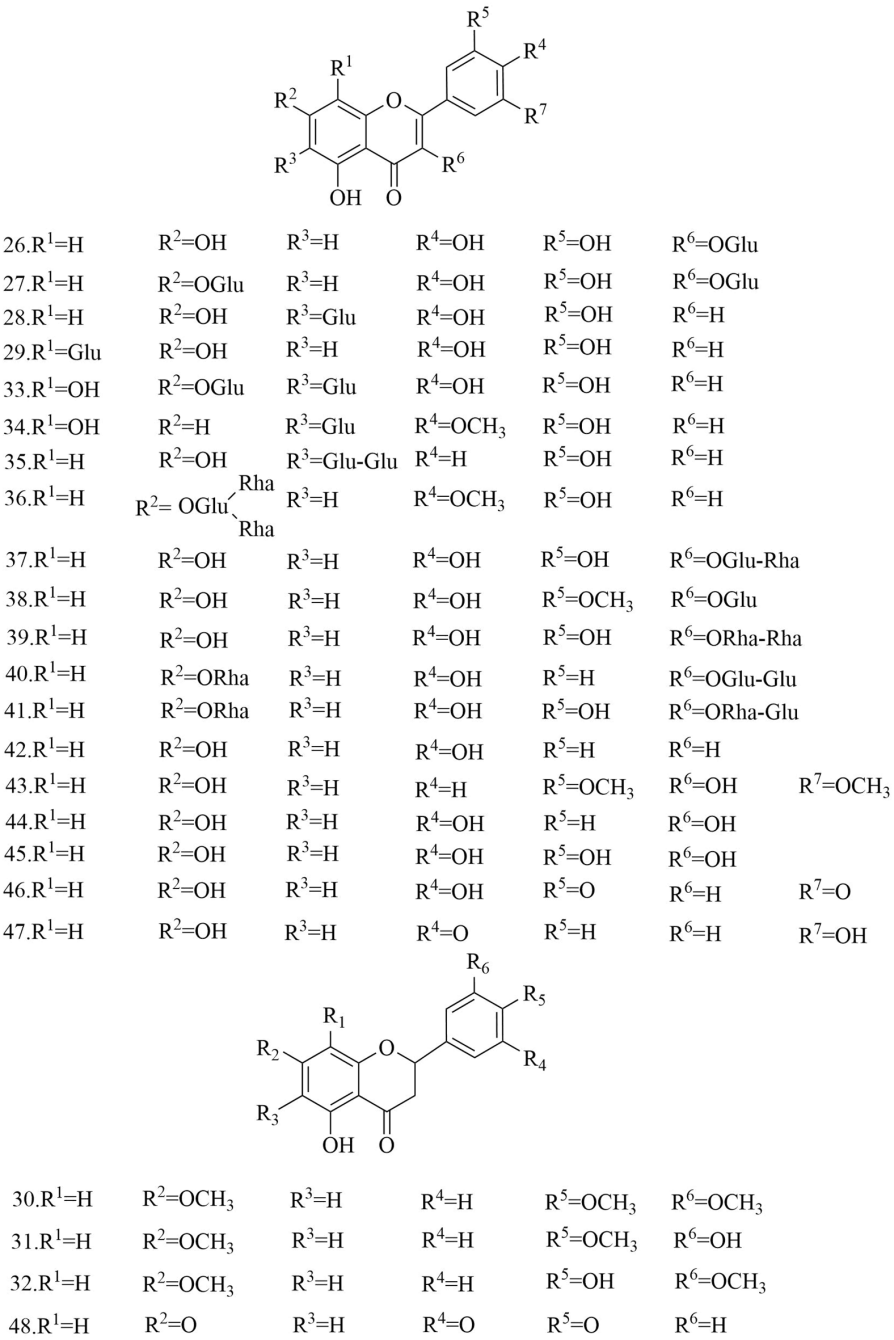
Chemical structures of flavonoids

**Fig. 7:**
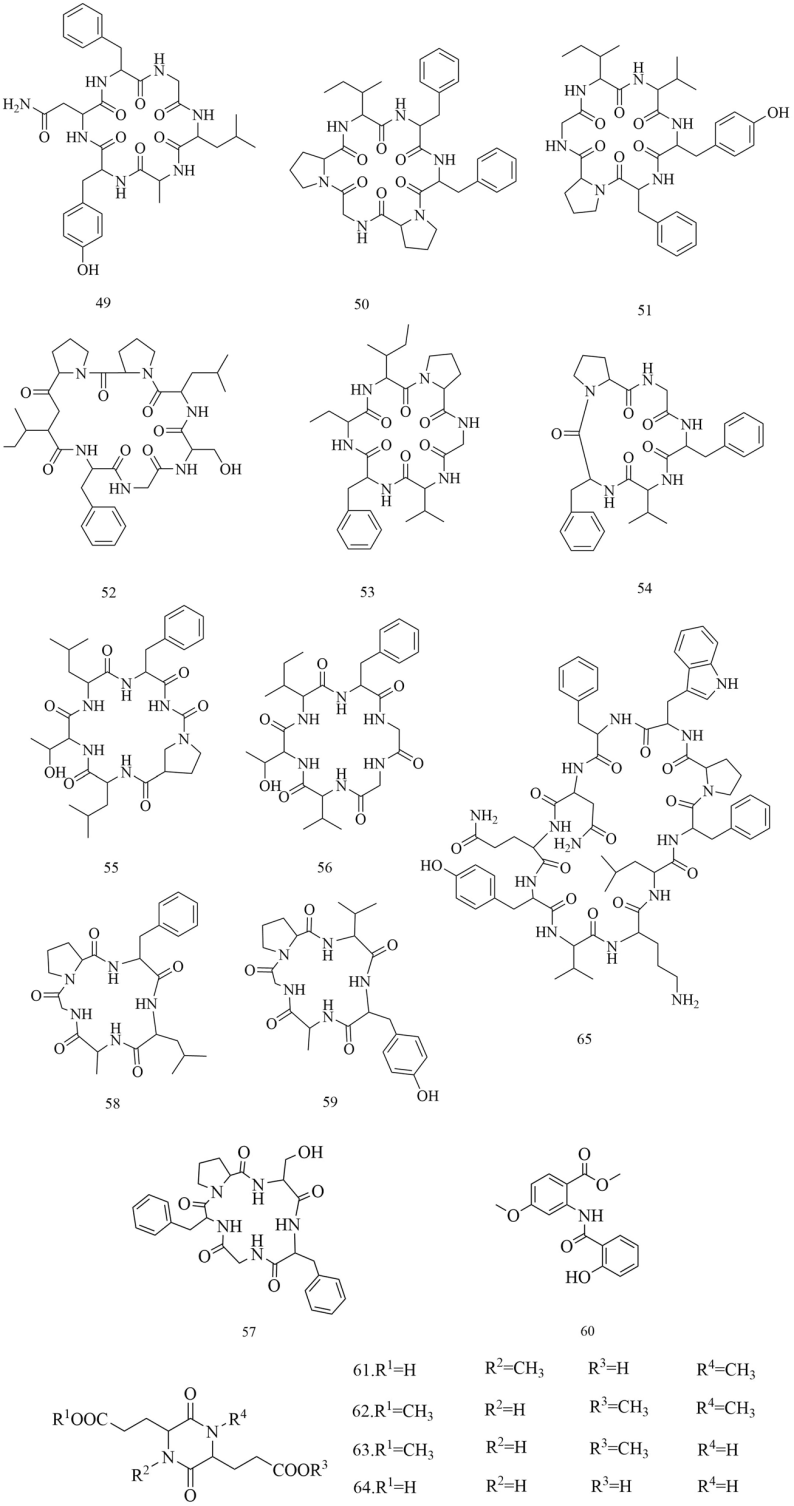
Chemical structures of peptides

**Fig. 8:**
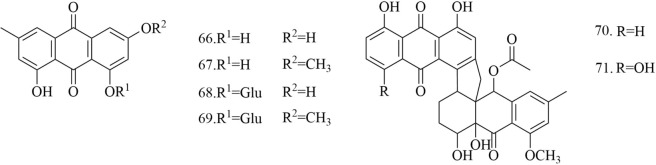
Chemical structures of anthraquinones

**Fig. 9:**
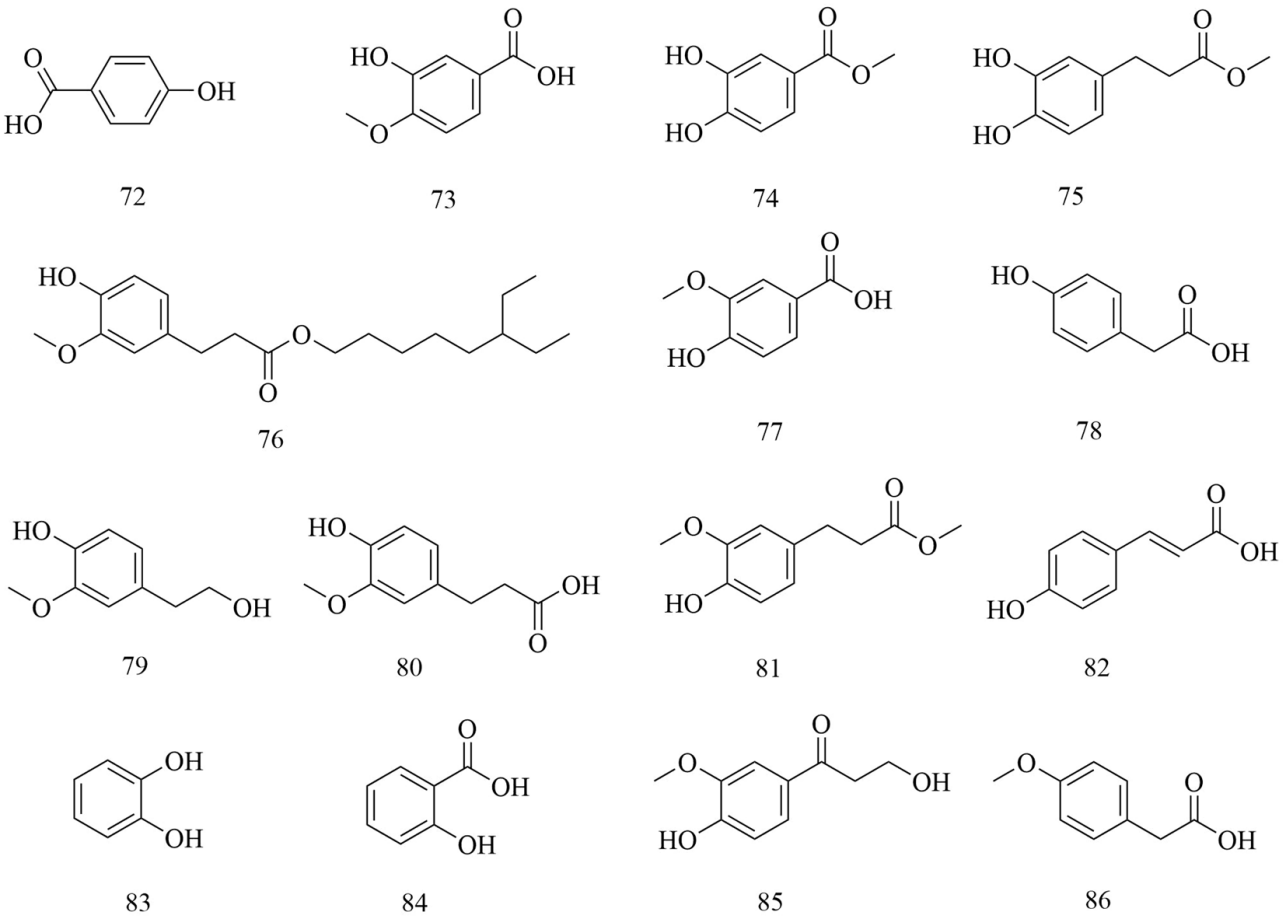
Chemical structures of phenolic acids

**Fig. 10:**
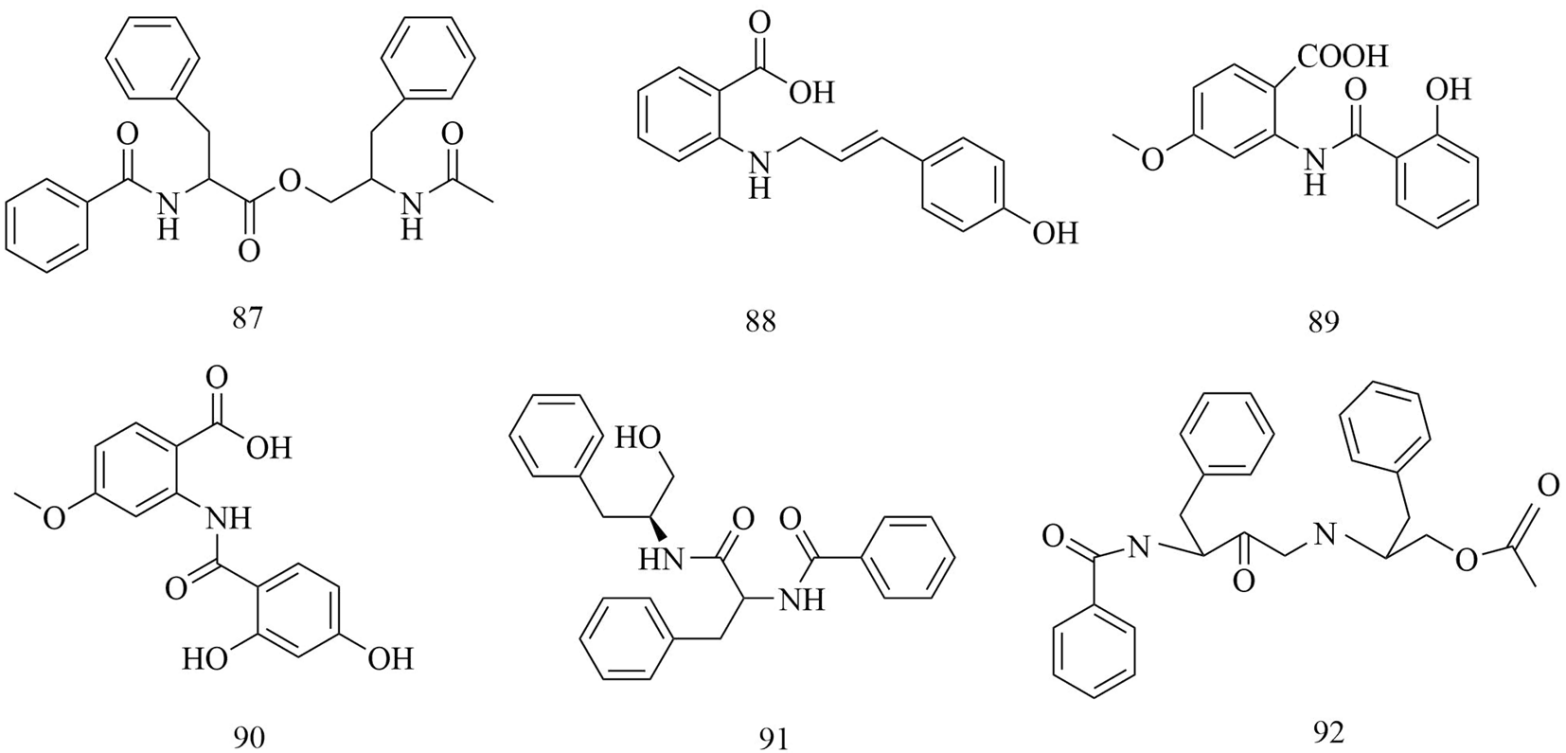
Chemical structures of amides

**Fig. 11:**
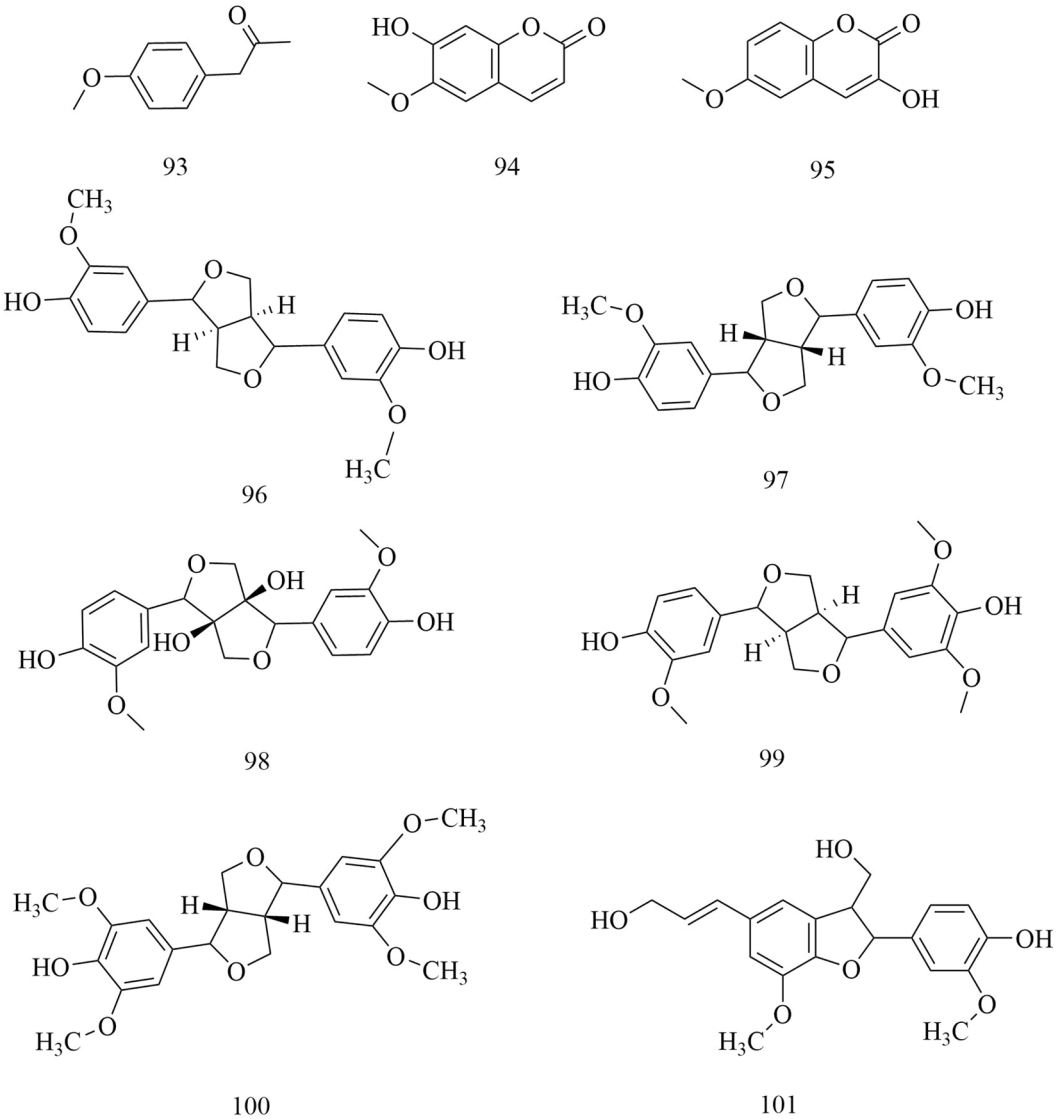
Chemical structures of phenylpropanoids

**Fig. 12:**
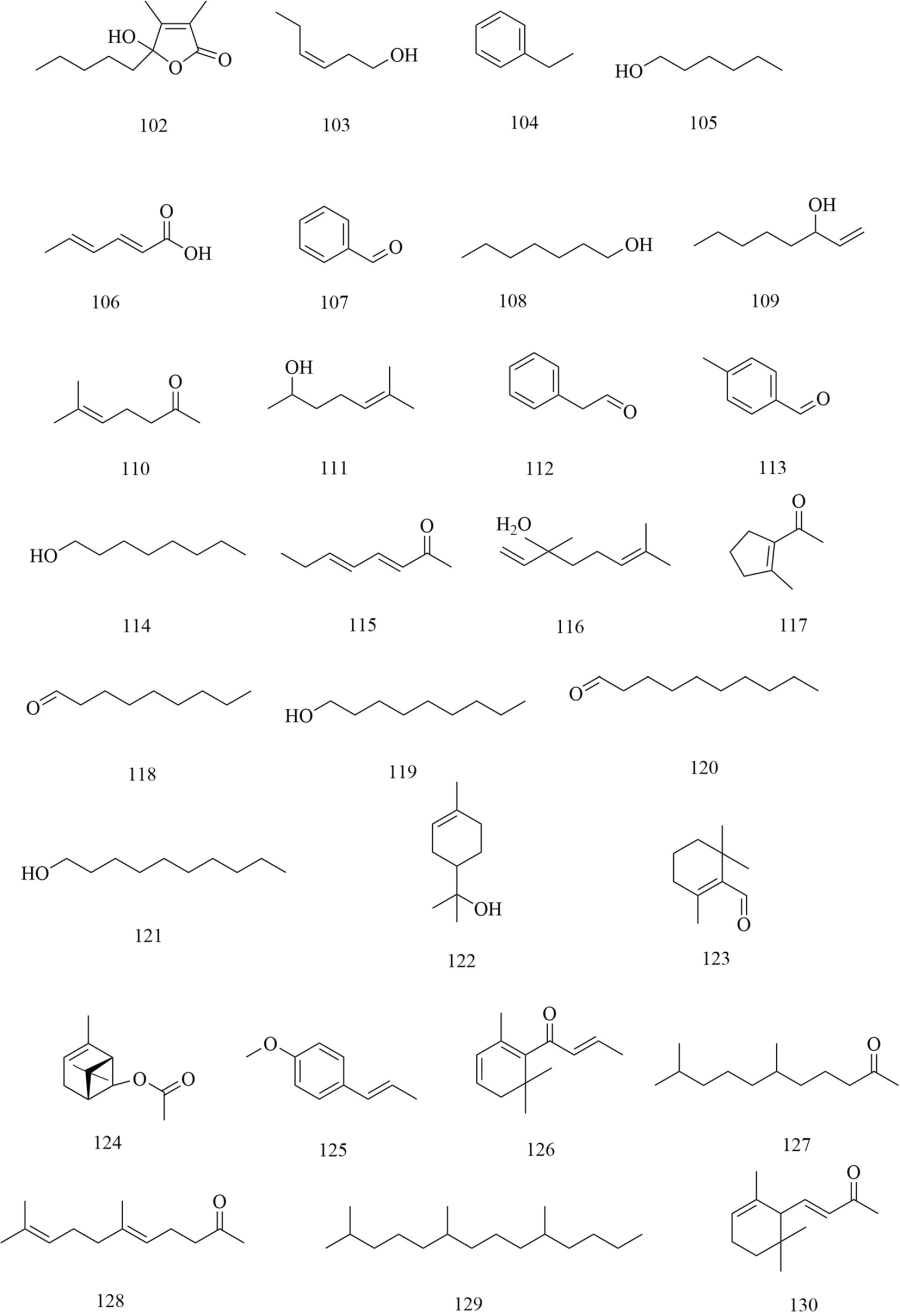

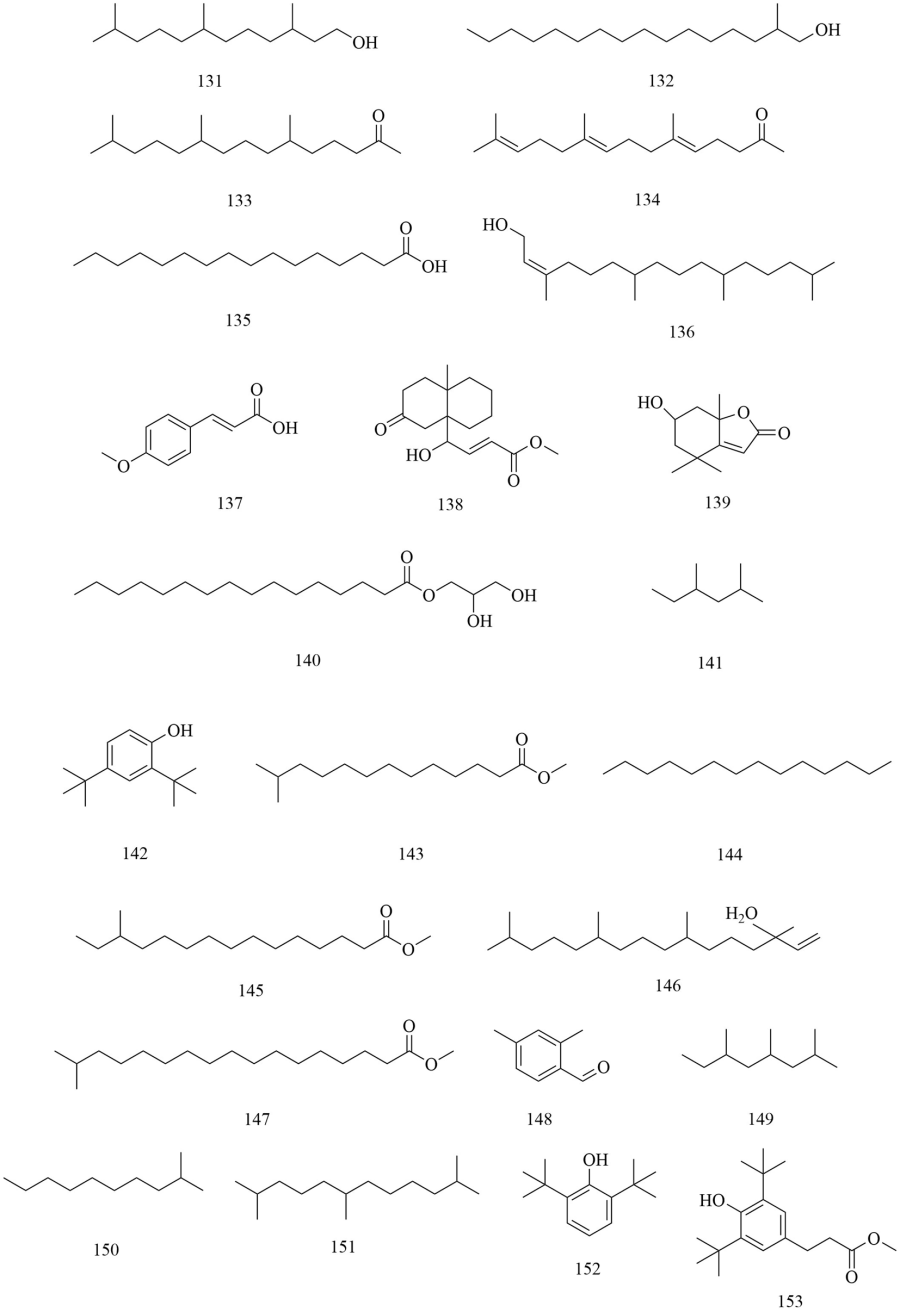

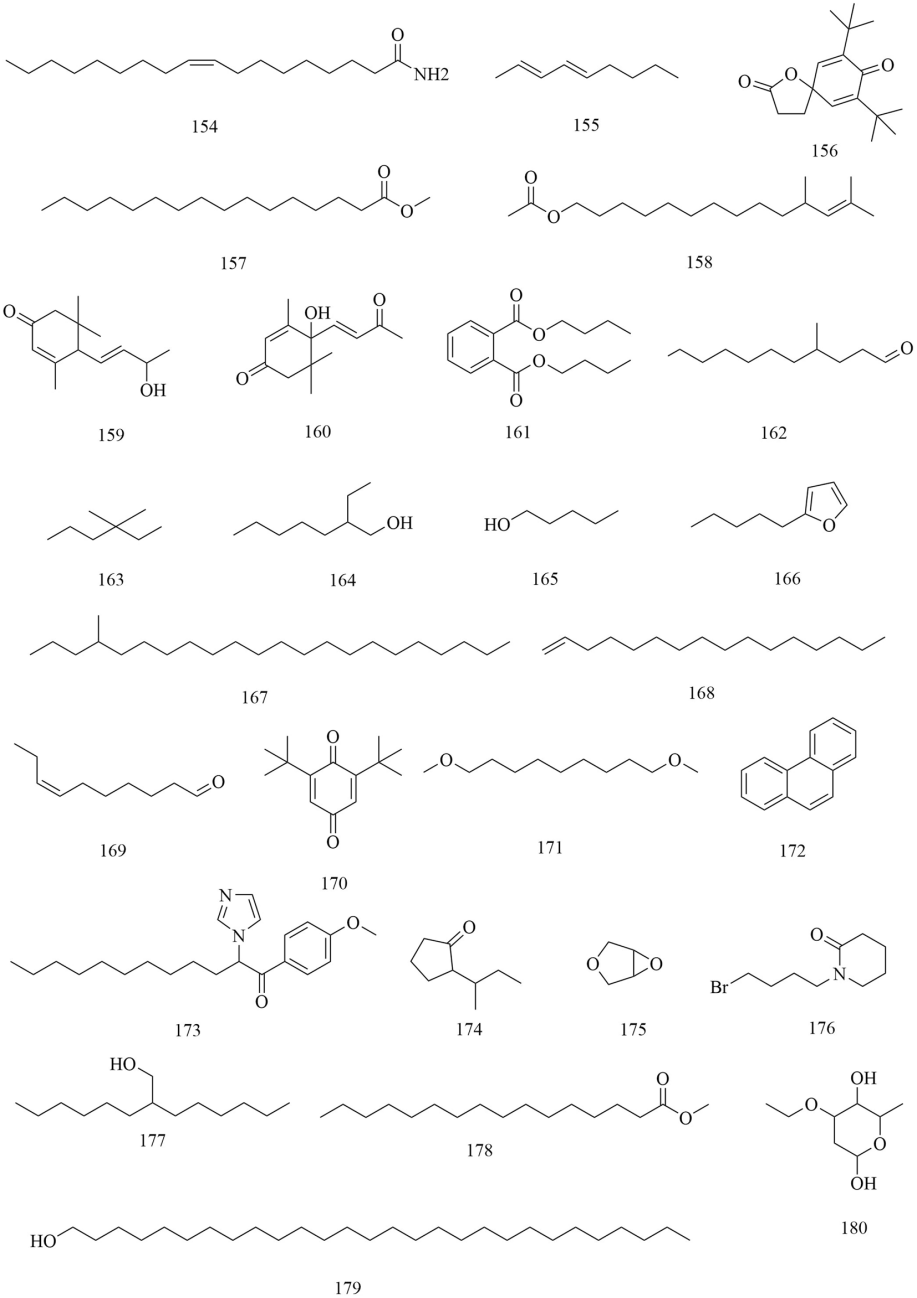

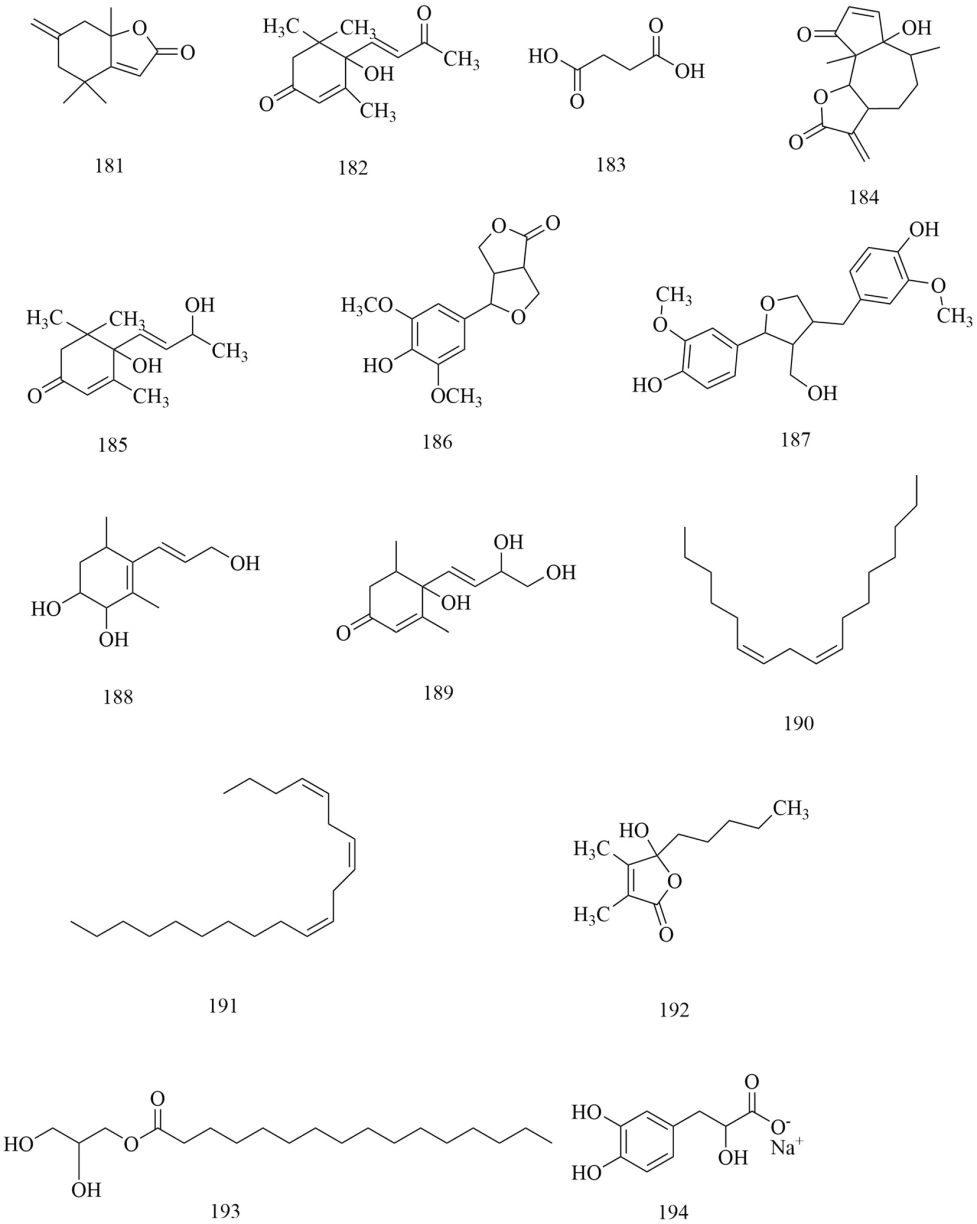
Chemical structures of others

**Table 3:** Compounds isolated from *D. superbus* and *D. chinensis*

Classification	No	Compounds	Source	Refs
Saponins	1	3-*O*-*β*-d-Glucopyranosylolean-9(11),12-diene-23,28-dioicacid28-*O*-*β*-d-glucopyranoside	*D. superbus*	[11]
	2	3-*O*-*β*-d-Glucopyranosylolean-11,13(18)-diene-23,28-dioicacid28-*O*-*β*-d-glucopyranoside	*D. superbus*	[11, 13]
	3	3-*O*-*β*-d-Glucopyranosyl gypsogenic acid 28-*O*-[*β*-d-6-*O*-((3*S*)-3-hydroxyl-3- methylglutaryl)glucopyranosyl(1 → 6)]-*β*-d-glucopyranoside	*D. superbus*	[12]
	4	3-*O*-*β*-d-Glucopyranosyl gypsogenic acid 28-*O-*[*β*-d-Glucopyranosyl(1 → 3)][*β*-d-6-*O*-((3*S*)-hydroxyl-3-methylglutaryl)glucopyranosyl(1 → 6)]*-β*-d-glucopyranoside	*D. superbus*	[12]
	5	3-*O*-*α*-l-Arabinopyranosyl-3*β*,16α-dihydroxyolean-12-en-23,28-dioic acid 28-*O*-[*β*-d-Glucopyranosyl-(1 → 6)]-*β*-d-glucopyranoside	*D. superbus*	[12]
	6	Asiatic acid	*D. superbus*	[14]
	7	Dianchinenoside A	*D. chinensis*	[15]
	8	Dianchinenoside B	*D. chinensis*	[15]
	9	Dianchinenoside C	*D. chinensis*	[17]
	10	Dianchinenoside D	*D. chinensis*	[17]
	11	Dianchinenoside E	*D. chinensis*	[16]
	12	Dianchinenoside F	*D. chinensis*	[16]
	13	Dianchinenoside G	*D. chinensis*	[16]
	14	Dianchinenoside H	*D. chinensis*	[16]
	15	3-*O*-*α*-l-Arabinopyranosyl hederagenin 28-*O*-*β*-d-Glucopyranosyl(1 → 6)-*β*-d-glucopyranoside	*D. chinensis*	[24]
	16	Madecassic acid	*D. superbus*	[19]
	17	Sterculin A	*D. superbus*	[18]
	18	(24*R*)-Cycloart-25-ene-3*β*,24-diol	*D. superbus*	[18]
	19	(24*S*)-Cycloart-25-ene-3*β*,24-diol	*D. superbus*	[18]
	20	*β*-Spinasterol	*D. superbus*	[18]
	21	Stigmast-7-en-3*β*-ol	*D. superbus*	[18]
	22	*β*-Sitosterol glucoside	*D. superbus*	[38]
	23	Hainanenside	*D. superbus*	[20]
	24	3-*O*-*β*-d-Glucopyranosyl olean-11, 13 (18)-diene-23, 28-dioic acid	*D. superbus*	[20]
	25	Quillaic acid	*D. superbus*	[21]
Flavonoids	26	Quercetin-3-*O*-glucoside	*D. superbus*	[22]
	27	Quercetin-7-*O*-glucoside	*D. superbus*	[27]
	28	Isoorientin	*D. superbus*	[23]
	29	Orientin	*D. superbus*	[23]
	30	5-Hydroxy-7,5′,4′-trimethoxyflavanone	*D. superbus*	[18]
	31	5,3′-Dihydroxy-7,4′-dimethoxyflavanone	*D. superbus*	[18]
	32	5,4′-Dihydroxy-7,3′-dimethoxyflavanone	*D. superbus*	[18]
	33	Isoorientin-2″-*O*-glucoside	*D. chinensis*	[24]
	34	Chrysoeriol-7-*O*-glucoside	*D. chinensis*	[24]
	35	Isovitexin-2″-*O*-glucoside	*D. chinensis*	[24]
	36	Diosmetin-7-*O*(2″,6″-di-*O*-α-l-rhamnopyranosyl)-*β*-d-glucopyranoside	*D. superbus*	[28, 42]
	37	Quercetin-3-*O*-rutinoside	*D. superbus*	[30]
	38	Isorhamnetin-3-*O*-glucoside	*D. superbus*	[30]
	39	Quercetin-3-*O*-rhamnoside 7-*O*-rhamnoside	*D. superbus*	[30]
	40	Kaempferol-3-*O*-glucoside-glucoside 7-*O*-rhamnoside	*D. superbus*	[30]
	41	Quercetin-3-*O*-rhamnoside-glucoside 7-*O*-rhamnoside	*D. superbus*	[30]
	42	Luteolin	*D. superbus D. chinensis*	[23, 25, 29]
	43	3,5,7-Trihydroxy-3′,5′-dimethoxylflavone	*D. superbus*	[26]
	44	Kaempferol	*D. superbus*	[26]
	45	Quercetrin	*D. superbus*	[26]
	46	Tricin	*D. superbus*	[31]
	47	Diosmetin	*D. superbus*	[31]
	48	5-Hydroxy-7,3',4'-trimethoxydihydroflavone	*D. superbus*	[20]
Peptides	49	Dianthin A	*D. superbus*	[33]
	50	Dianthin B	*D. superbus*	[33]
	51	Dianthin C	*D. superbus*	[34]
	52	Dianthin D	*D. superbus*	[34]
	53	Dianthin E	*D. superbus*	[34]
	54	Dianthin F	*D. superbus*	[34]
	55	Dianthin G	*D. superbus*	[35]
	56	Dianthin H	*D. superbus*	[35]
	57	Dianthin I	*D. chinensis*	[37, 45]
	58	Pseudostellarin A	*D. chinensis*	[37, 45]
	59	Heterophyllin J	*D. chinensis*	[37, 45]
	60	4-Methoxydianthramide B	*D. superbus*	[34]
	61	Cyclo-(l-*N*-methyl Glu-l-*N*-methyl Glu	*D. chinensis*	[36]
	62	Cyclo-(l-methyl Glu-ester-l-methyl Glu ester	*D. chinensis*	[36]
	63	Cyclo-(l-methyl Glu-ester-l-Glu)	*D. chinensis*	[36]
	64	Cyclo-(l-Glu-l-Glu)	*D. chinensis*	[36]
	65	Tyrocidine B	*D. superbus*	[30]
Anthraquinones	66	Emodin	*D. superbus*	[19, 25, 38, 40]
	67	Physcion	*D. superbus*	[38, 39]
	68	Emodin-8-*O*-*β*-d-glucopyranoside	*D. superbus*	[25, 38]
	69	Physcion-8-*O*-*β*-d-glucoside	*D. superbus*	[20]
	70	MelrubiellinA	*D. superbus*	[21]
	71	MelrubiellinB	*D. superbus*	[21]
Phenolic acids	72	*p*-Hydroxybenzoic acid	*D. superbus*	[19, 41]
	73	3-Hydroxy-4-methoxybenzoic acid	*D. superbus*	[19]
	74	Methyl 3,4-dihydroxybenzoate	*D. superbus*	[38]
	75	Methyl 3-(3,4-dihydroxyphenyl) propionate	*D. superbus*	[38]
	76	4-hydroxy-3-methoxy-pentyl ester benzenepropanoic acid	*D. superbus*	[42]
	77	Vanillic acid	*D. superbus*	[42]
	78	4-Hydroxy-benzeneacetic acid	*D. superbus*	[42]
	79	Hydroferulic acid	*D. superbus*	[42]
	80	Methyl hydroferulate	*D. superbus*	[42]
	81	(*E*)-methyl-4-hydroxy-4-(8a-methyl-3-oxodecahydronaphthalen-4a-yl)	*D. superbus*	[42]
	82	Trans-*p*-coumaric acid	*D. superbus*	[26]
	83	Catechol	*D. superbus*	[21]
	84	Salicylic acid	*D. superbus*	[21]
	85	*β*⁃hydroxypropiovanillone	*D. superbus*	[31]
	86	4-Methoxyphenylacetic acid	*D. superbus*	[20]
Amides	87	Patriscabratine	*D. superbus*	[19]
	88	*N*-*p*-coumarylanthranilic acid	*D. superbus*	[19]
	89	Methoxydianthramide S	*D. superbus*	[26]
	90	2-[(2,4-Dihydroxybenzoyl) amino]-4-methoxy-benzoic acid	*D. superbus*	[26]
	91	Aurantiamide	*D. superbus*	[21]
	92	Aurantiamide acetate	*D. superbus*	[31]
Phenylpropanoids	93	4-Methoxy-benzeneacetic acid	*D. superbus*	[42]
	94	Scopoletin	*D. superbus*	[26]
	95	6-Methoxy-hydroxycoumarin	*D. superbus*	[26]
	96	Epipinoresinol	*D. superbus*	[21]
	97	Pinoresinol	*D. superbus*	[21]
	98	Prinsepiol	*D. superbus*	[21]
	99	Medioresinol	*D. superbus*	[31]
	100	Syringaresinol	*D. superbus*	[31]
	101	Dehydrodiconiferyl alcohol	*D. superbus*	[31]
Others	102	Hydoxydihydrobovolide	*D. superbus*	[18]
	103	Cis-3-hexen-1-ol	*D. superbus*	[43]
	104	Phenylethane	*D. superbus*	[43]
	105	*N*-hexanol	*D. superbus*	[43]
	106	Sorbic Acid	*D. superbus*	[43]
	107	Benzaldehyde	*D. superbus*	[43]
	108	*N*-heptanol	*D. superbus*	[43]
	109	1-Octen-3-ol	*D. superbus*	[43]
	110	6-Methyl-5-hepten-2-one	*D. superbus*	[43]
	111	2-Methyl-2-hepten-6-ol	*D. superbus*	[43]
	112	Phenylacetaldehyde	*D. superbus*	[43]
	113	*p*-tolualdehyde	*D. superbus*	[43]
	114	*N*-octanol	*D. superbus*	[43]
	115	(*E*, *E*)-3,5-octadien-2-one	*D. superbus*	[43]
	116	Linalool	*D. superbus*	[43]
	117	1-Acetyl-2-methylcyclopentene	*D. superbus*	[43]
	118	*N*-nonanal	*D. superbus*	[43]
	119	*N*-nonanol	*D. superbus*	[43]
	120	decanal	*D. superbus*	[43]
	121	*n*-decanol	*D. superbus*	[43]
	122	*α*-terpineol	*D. superbus*	[43]
	123	*α*-cyclocitral	*D. superbus*	[43]
	124	Cis-chrysanthenylacetate	*D. superbus*	[43]
	125	(*E*)-anethole	*D. superbus*	[43]
	126	*α*-Damascenone	*D. superbus*	[43]
	127	Tetrahydrogeranyl acetone	*D. superbus*	[43]
	128	Geranyl acetone	*D. superbus*	[43]
	129	2,6,10-trimethyltetradecane	*D. superbus*	[43]
	130	*β*-Ionone	*D. superbus*	[43]
	131	3,7,11-Trimethyl-1-dodecanol	*D. superbus*	[43]
	132	2-Methylhexadecan-1-ol	*D. superbus*	[43]
	133	6,10,14-Trimethyl-2-pentadecanone	*D. superbus*	[43]
	134	Farnesy lacetone	*D. superbus*	[43]
	135	Palmitic acid	*D. superbus*	[43]
	136	Cis-phytol	*D. superbus*	[43]
	137	(*E*)-4-Methoxycinnamic acid	*D. superbus*	[42]
	138	3-Methoxy-4-hydroxyphenylethanol	*D. superbus*	[42]
	139	Loliolide	*D. superbus*	[26]
	140	1-Monopalmitin	*D. superbus*	[26]
	141	2,4-Dimethylhexane	*D. superbus*	[44]
	142	2,4-Di-tert-butylphenol	*D. superbus*	[44]
	143	Methyl 12-methyltridecanoate	*D. superbus*	[44]
	144	Tetradecane	*D. superbus*	[44]
	145	Methyl 13-methylpentadecanoate	*D. superbus*	[44]
	146	3,7,11,15-Tetramethyl-1-hexadecadiene-3-ol	*D. superbus*	[44]
	147	16-methylheptadecanoic acid methyl ester	*D. superbus*	[44]
	148	2,4-dimethylbenzaldehyde	*D. superbus*	[44]
	149	2,4,6-trimethyloctane	*D. superbus*	[44]
	150	2-Methyldecane	*D. superbus*	[44]
	151	2,6,11-trimethyldodecane	*D. superbus*	[44]
	152	2,6-di-tert-butylphenol	*D. superbus*	[44]
	153	Methyl-3-(3,5-di-tert-butyl-4-hydroxyphenyl) propionate	*D. superbus*	[44]
	154	(*Z*)-9-Octadecenylamide	*D. superbus*	[44]
	155	2,4-Nondiene	*D. superbus*	[44]
	156	7,9-Di-tert-butyl-1-oxaspiro (4,5) deca-6,9-diene-2,8-dione	*D. superbus*	[44]
	157	Methyl palmitate	*D. superbus*	[44]
	158	11,13-dimethyl-12-tetradecene-1-ol acetate	*D. superbus*	[44]
	159	(*R*)-4-[(*R*, *E*)-3-hydroxy-1-butenyl]-3,5,5-trimethyl-2-cyclohexene-1-one	*D. superbus*	[44]
	160	1-Hydroxy-4-keto-2-ionone	*D. superbus*	[44]
	161	Dibutyl phthalate	*D. superbus*	[44]
	162	4-Methyl-1-undecanone	*D. superbus*	[44]
	163	3, 3-Dimethylhexane	*D. superbus*	[44]
	164	2-Ethyl-1-heptanol	*D. superbus*	[44]
	165	1-Pentanol	D. superbus	[44]
	166	2-n-Pentylfuran	*D. superbus*	[44]
	167	4-Methyldocosane	*D. superbus*	[44]
	168	1-Hexadecene	*D. superbus*	[44]
	169	Cis-7-decene aldehyde	*D. superbus*	[44]
	170	2,6-Di-tert-butyl-p-benzoquinone	*D. superbus*	[44]
	171	Dimethyl azelaite	*D. superbus*	[44]
	172	Phenanthrene	*D. superbus*	[44]
	173	2-(1H-imidazol-1-yl)-1-(4-methoxyphenyl)-1-dodecanone	*D. superbus*	[44]
	174	2-Sec-butyl Cyclopentanone	*D. superbus*	[44]
	175	3,4-Epoxytetrahydrofuran	*D. superbus*	[44]
	176	2-Oxo-1-(4-bromo-*n*-butyl) piperidine	*D. superbus*	[44]
	177	2-Hexyl-1-octanol	*D. superbus*	[44]
	178	Methyl hexadecanoate	*D. superbus*	[44]
	179	1-Octacosanol	*D. superbus*	[44]
	180	l-Dianose	*D. chinensis*	[44]
	181	Dehydrololiolide	*D. superbus*	[21]
	182	Dehydrovomifoliol	*D. superbus*	[21]
	183	Succinic acid	*D. superbus*	[21]
	184	Parthenin	*D. superbus*	[31]
	185	Vomifoliol	*D. superbus*	[31]
	186	Zhebeiresinol	*D. superbus*	[31]
	187	Lariciresinol	*D. superbus*	[31]
	188	Ixerol B	*D. superbus*	[31]
	189	Cucumegastigmanes I	*D. superbus*	[31]
	190	(6z, 9z)-heptadecadiene	*D. superbus*	[31]
	191	3, 6, 9-nonadecatriene	*D. superbus*	[21]
	192	5-Hydroxyl-3,4-dimethy-5-pentyl-2(5H)-furanone	*D. superbus*	[20]
	193	1-Glycerol palmitate	*D. superbus*	[20]
	194	3-(3',4'-Dihydroxyphenyl)lactic acid sodium salt	*D. superbus*	[20]

### Saponins

Saponins are one of the main chemical constituents of *D. superbus* and *D. chinensis*. Oleanane-type triterpenoid saponins have been identified as characteristic constituents of these plants [10]. Nineteen triterpenoid and three steroid saponins have been isolated and identified in *D. superbus* and *D. chinensis.* The triterpenoid saponins 12-diene-23,28-dioic acid 28-*O*-*β*-d-glucopyranoside (**1**), 3-*O*-*β*-d-glucopyranosyl olean-11,13(18)-diene-23,28-dioic acid 28-*O*-*β*-d-glucopyranoside (**2**), 3-*O*-*β*-d-glucopyranosyl olean-28-*O*-[*β*-d-*O*-((3S)-3-hydroxyl-3-methlglutaryl)-glucopyranosyl-(1 → 6)]-*β*-d-glucopyranoside (**3**), 3-*O*-*β*-d-glucopyranosyl gypsogenic acid 28-*O*-[*β*-d-glucopyranosyl(1 → 3)][*β*-d-6-((3S)-hydroxyl-3-2methlglutaryl)-glucopyranosyl-(1 → 6)]-*β*-d-glucopyranoside (**4**), 3-*O*-α-l-arabinopyranosyl-3b,16a-dihydroxyolean-12-en-23,28-dioic acid 28-*O*-[*β*-d-glucopyranosyl-(1 → 6)]-*β*-d-glucopyranoside (**5**), and asiatic acid (**6**) have been isolated from the dried aerial parts of *D. superbus* [11–14]. Four triterpenoid saponins, dianchinenoside A-H (**7–14**) were isolated from the dried aerial parts of *D. chinensis* [15, 16]. The compound, 3-*O*-*α*-l-arabinopyranosyl hederagenin 28-*O*-*β*-d-glucopyranosyl(1 → 6)-*β*-d-glucopyranoside (**15**) was isolated from the dried aerial parts of *D. chinensis* [17]. The triterpenoid saponins madecassic acid (**16**), sterculin A (**17**), (24*R*)-cycloart-25-ene-3*β*,24-diol (**18**), and (24*S*)-cycloart-25-ene-3*β*,24-diol (**19**) were isolated from the dried aerial parts of *D. superbus* [18, 19]. Steroid saponins including *β*-spinasterol (**20**), stigmast-7-en-3*β*-ol (**21**), *β*-sitosterol glucoside (**22**), Hainanenside (**23**), 3-*O-β*-d-glucopyranosyl olean-11, 13 (18)-diene-23, 28-dioic acid (**24**) and Quillaic acid (**25**) have been isolated from the dried aerial parts of *D. superbus* [20–22] (Fig. 5).

### Flavonoids

Flavonoids are an important class of natural organic compounds with a basic 2-phenyl-chromone structure; they are widely distributed in the plant kingdom. Most natural flavonoids exist in the form of glycosides, which differ in composition according to the type, quantity, linkage position, and connection mode of the sugar. A few flavonoids and flavonoid glycosides have been reported in *D. superbus* and *D. chinensis.*

To date, the following 20 flavonoids have been isolated from these plants, including quercetin-3-*O*-glucoside (**26**), quercetin-7-*O*-glucoside (**27**), isoorientin (**28**), orientin (**29**), 5-hydroxy 7,3′,4′-trimethoxyflavanone (**30**), 5,3′-dihydroxy-7,4′-dimethoxyflavanone (**31**), 5,4′-dihydroxy-7,3′-dimethoxyflavanone (**32**), isoorientin-2′-*O*-glucoside (**33**), chrysoeriol-7-*O*-glucoside (**34**), isovitexin-2″-*O*-glucoside (**35**), diosmetin-7-*O*(2″,6″-di-*O*-*α*-l-rhamnopyranosyl)-*β*-d-glucopyranoside (**36**), quercetin-3-*O*-rutinoside (**37**), isorhamnetin-3-*O*-glucoside (**38**), quercetin-3-*O*-rhamnoside 7-*O*-rhamnoside (**39**), kaempferol-3-*O*-glucoside-glucoside 7-*O*-rhamnoside (**40**), quercetin-3-*O*-rhamnoside-glucoside 7-*O*-rhamnoside (**41**), luteolin (**42**), 3,5,7-trihydroxy-3′, 5′-dimethoxylflavone (**43**), kaempferol (**44**), quercetrin (**45**), Tricin (**46**), Diosmetin (**47**) and 5-Hydroxy-7,3',4'-trimethoxydihydroflavone (**48**) [18, 20, 22–31] (Fig. 6).

### Peptides

Cyclic peptides are a class of peptides with a variety of applications and have been widely studied over the past decades [32]. Cyclic peptides were first isolated from *D. superbus* in 1998, and they have gradually become a hotspot in chemical research [33]. Ten peptides namely dianthin A-H **(49–56**), 4-methoxydianthramide B (**60**), and tyrocidine B (**65**) were obtained from *D. superbus* [30, 33–35]*.* The seven peptides in *D. chinensis* are dianthin I (**57**), pseudostellarin A (**58**), heterophyllin J (**59**), cyclo-(l-*N*-methyl Glu-l-*N*-methyl Glu (**61**), cyclo-(l-methyl Glu-ester-l-methyl Glu ester (**62**), cyclo-(l-methyl Glu-ester-l-Glu) (**63**), and cyclo-(l-Glu-l-Glu) (**64**) [36, 37] (Fig. 7).

### Anthraquinones

Three anthraquinones, emodin (**66**), physcion (**67**), emodin-8-*O*-*β*-d-glucopyranoside (**68**), Physcion-8-*O-β*-d-glucoside (**69**), melrubiellinA (**70**) and melrubiellinB (**71**) have been isolated from *D. superbus* and *D. chinensis* [19–21, 25, 38–40] (Fig. 8).

### Phenolic acids

A total of 15 phenolic acids were isolated. Including *p*-Hydroxybenzoic acid (72), 3-Hydroxy-4-methoxybenzoic acid (73), Methyl 3,4-dihydroxybenzoate (74), Methyl 3-(3,4-dihydroxyphenyl) propionate(75), 4-hydroxy-3-methoxy-pentyl ester benzenepropanoic acid (76), Vanillic acid (77), 4-Hydroxy-benzeneacetic acid (78), Hydroferulic acid (79), Methyl hydroferulate (80), (E)-methyl-4-hydroxy-4-(8a-methyl-3-oxodecahydronaphthalen-4a-yl) (81), Trans-*p*-coumaric acid (82), Catechol (83), Salicylic acid (84), β⁃hydroxypropiovanillone (85) and 4-Methoxyphenylacetic acid (86) [19–21, 26, 31, 38, 41, 42] (Fig. 9).

### Amides

Amides are also found in *D. superbus* and *D. chinensis*. Both Patriscabratine (87), *N*-*p*-coumarylanthranilic acid (88), Methoxydianthramide S (89), 2-[(2,4-Dihydroxybenzoyl) amino]-4-methoxy-benzoic acid (90), Aurantiamide (91) and Aurantiamide acetate (92) are reported compounds [19, 21, 26, 31] (Fig. 10).

### Phenylpropanoids

Phenylpropanol is a kind of phenolic substance that exists naturally. The special phenylpropa-noid compounds found in *D. superbus* and *D. chinensis* are 4-Methoxy-benzeneacetic acid (93), Scopoletin (94), 6-Methoxy-hydroxycoumarin (95), epipinoresinol (96), Pinoresinol (97), Prinsepiol (98), Medioresinol (99), Syringaresinol (100) and Dehydrodiconiferyl alcohol (101) [21, 26, 31, 42] (Fig. 11).

### Others

In addition to the compounds mentioned above, a variety of volatile oils and other compounds, including aromatic and aliphatic compounds, have also been isolated and identified. The compound hydroxydihydrobovolide (**102**) has been isolated from the dried aerial parts of *D. superbus* and extracted and comprehensively analyzed in large amounts from the volatile oils of *D. superbus* using gas chromatography-mass spectrometry (GC–MS) [18, 19]: cis-3-hexen-1-ol (**103**), phenylethane (**104**), *n*-hexanol (**105**), sorbic acid (**106**), benzaldehyde (**107**), *n*-heptanol (**108**), 1-octen-3-ol (**109**), 6-methyl-5-hepten-2-one (**110**), 2-methyl-2-hepten-6-ol (**111**), phenylacetaldehyde (**112**), *p*-tolualdehyde (**113**), *n*-octanol (**114**), (*E*,*E*)-3,5-octadien-2-one (**115**), linalool (**116**), 1-acetyl-2-methylcyclopentene (**117**), *n*-nonanal (**118**), *n*-nonanol (**119**), decanal (**120**), *n*-decanol (**121**), *α*-terpineol (**122**), *α*-cyclocitral (**123**), *cis*-chrysanthenyl acetate (**124**), (*E*)-anethole (**125**), *α*-damascenone (**126**), tetrahydrogeranyl acetone (**127**), geranyl acetone (**128**), 2,6,10-trimethyltetradecane (**129**), *β*-ionone (**130**), 3,7,11-trimethy l-1-dodecanol (**131**), 2-methylhexadecan-1-ol (**132**), 6,10,14-trimethyl-2-pentadecanone (**133**), farnesyl acetone (**134**), palmitic acid (**135**), and cis-phytol (**136**). These components account for 73.45% of the total volatile oils in *D. superbus.* The results showed that the main components of the essential oils are 6,10,14-trimethyl-2-pentadecanone (28.39%), cis-phytol (6.80%), geranyl acetone (4.65%), n-hexanol (4.32%), and farnesyl acetone (3.01%) [43]. (*E*)-4-methoxycinnamic acid (**137**), 3-methoxy-4-hydroxyphenylethanol (**138**), loliolide (**139**), and 1-monopalmitin (**140**) were isolated from the dried aerial parts of *D. superbus* [26, 38, 42]. The compounds (**141–179**) were identified by GC–MS in the petroleum ether extract from *D. superbus* [44]. The monosaccharide l-Dianose (**180**) was identified by ^1^H-NMR (nuclear magnetic resonance) and ^13^C-NMR data from the aerial parts of *D. chinensis* [24]. Other compounds including Dehydrololiolide (**181**), Dehydrovomifoliol (**182**), Succinic acid (**183**), Parthenin (**184**), Vomifoliol (**185**), Zhebeiresinol (**186**), Lariciresinol (**187**), ixerol B (**188**), Cucumegastigmanes I (**189**), (6z, 9z)-heptadecadiene (**190**), 3, 6, 9⁃nonadecatriene (**191**), 5-hydroxyl-3,4-dimethy-5-pentyl-2(5H)-furanone (**192**), 1-glycerol palmitate (**193**) and 3-(3',4'-Dihydroxyphenyl)lactic acid sodium salt (**194**) were also found in *D. superbus* [20, 21, 31] (Fig. 12).

## Pharmacology

Dianthi herba has a long history of medicinal use and has been reported to have various pharmacological activities, including antitumor [46], antioxidant [47], antiviral [48], anti-inflammatory [49, 50], diuretic [51], uterine excitatory [52], antimicrobial [53], and neuroprotective activity [42]. These activities are summarized in Table 4.

**Table 4:** The pharmaceutical effects of Dianthi

Pharmaceutical effects	Compounds/extracts	Doses	Models (in vivo)	Models (in vitro)	Results/mechanism	Refs
Anti-tumor	Asiatic acid	55.4, 48.9, 55.3, 58.8 μg/mL	–	SW480, MCF-7, MDA-MB-231, SK-MEL-2, HCT116, T98G, SSMC-7721, SK-HEP-1, and Bel-7402 cell	Activating the mitochondrial apoptosis pathway, which leads to apoptosis; Regulating Pdcd4 through PI3K/Akt/mTOR/p70S6K signal transduction pathway to inhibit proliferation, migration and induced apoptosis of cancer cells; Induce apoptosis by inhibiting autophagy	[14, 121–123]
	Ethanol extract	180 μg/mL	–	Hep G2 cell	Downregulating the expression of bcl-xl and bcl-2, induces apoptosis through the mitochondrial pathway and caspase activation in HepG2 cells	[56]
	Ethanol extract	40, 60 μg/mL	–	SCC-15 and YD-15 oral cancer cells	Inhibited cell growth and induced apoptosis in both cell lines by decreasing the expression of Sp1 and Mcl-1	[57]
	Petroleum ether extract	100 mg/L	–	Hela, Smmc-7721, Hep G2, SK-HEP1, A549, and Bel-7402 cell	By regulating the expression of tumor-related factors and genes, changing the characteristics of mitochondria, affecting the cell cycle, inducing the differentiation of tumor cells, changing the calcium pump on the tumor membrane, reducing the activity of some enzymes needed for cell growth, and inhibiting the growth of tumor cells	[44, 124, 125]
Antioxidant	Dunhuang Yifang Dianthi Herba decoction	5, 10, 20 g/kg	Kidney calcium oxalate calculus in rats	–	By activating the Nrf2/ARE signal pathway and the expression of its downstream target genes NQO1 and SOD, it can enhance the antioxidation and further inhibit the formation of calcium oxalate stones	[58]
	Total flavonoids	0.065, 0.046 g/L	–	–	Exert antioxidant activity by directly scavenging free radicals, inhibiting oxidase and chelating metal ions	[60]
	Dianthi Herba decoction	3.13 mg/mL	Wistar rat	–	Decreasing the concentration of serum Ca2 + and up-regulate the expression of functional proteins related to Nrf2/NQO1 pathway, and improve renal oxidative stress injury	[61]
	Ethyl acetate extract	1.25 mg/mL	–	–	Significantly inhibiting the production of DPPH free radical and ABTS free radical	[62]
Antiviral	Quercetin 3-glucoside	4.93, 6.43, 9.94, 8.3, and 7.1 μg/mL	–	Influenza A/PR/8/34, A/Victoria/3/75, A/WS/33, B/Maryland/1/59, and B/Lee/40 viruses strains	Inhibiting virus-induced ROS production and AVO formation, block virus replication	[22]
	Quercetin-7-O-glucoside	3.1, 6.61, 8.19, and 5.17 μg/mL	–	Influenza A/PR/8/34, A/Vic/3/75, B/Lee/40, and B/Maryland/1/59 virus strains	Inhibiting virus-induced ROS and vesicle organelle formation, block viral RNA polymerase PB2, and inhibit viral RNA replication	[27]
Anti-inflammatory	Ethanolic extract	200 mg/kg	Ovalbumin-induced murine model of asthma	–	Reducing airway inflammation induced by OVA in asthmatic mice by down-regulating the expression of iNOS	[65]
	Dichloromethane-soluble fraction	50 μg/mL	–	Human alloreactive T cell	Blocking Akt phosphorylation of T cells; Prevent Foxp3 transcription	[67]
	Lipophilic organic acids and intermediate lipophilic organic acids from aqueous extracts	20 μg/mL	-	Human B cell line, Human myeloma cells	Markedly suppressed IgE production	[68]
Uterine excitatory activity	Fruit extracts of *D. superbus*	8.5, 17, and 34 g/kg	Early pregnancy in mice	–	Reducing the level of progesterone and block the normal development of decidua	[75]
	Methyl 3,4-dihydroxybenzoate	200 μg/kg	Mouse (pregnancy)	–	Stimulating the muscle strips of pregnant uterus and cooperate with oxytocin to enhance the intensity and amplitude of spontaneous contraction of pregnant uterus	[38]
Antimicrobial	Dianthi Herba decoction	2 mg/mL	–	*Chlamydia trachomatis*	The volume and quantity of *Chlamydia inclusions* decreased gradually	[78]
	Cyclopolypeptide	6 µg/mL	–	*Candida albicans*	Exhibited potent activity against the pathogenic fungus, *Candida albicans*, with a MIC concentration of 6 µ g/mL	[79]
	Ethanolic extract; Water extract	6.25–12.5 mg/mL; 12.5–50 mg/mL	–	*Shigella dysenteriae*, *Bacillus cereus*, and *Vibrio cholerae*	It has significant inhibitory activity against three bacteria, and the MIC range is 6.25–12.5 mg/mL and 12.5–50 mg/mL respectively	[96]

### Antitumor activity

Studies have shown that the triterpenoids, cyclic peptides, flavonoids, and other components present in *Dianthus* have antitumor activity [54, 55]. Zhang et al. used the 3-(4,5-dimethylthiazol-2-yl)-2,5-diphenyltetrazolium bromide (MTT) assay to identify and study the antitumor active ingredients in *Dianthus.* The active ingredient asiatic acid was obtained from the n-butanol site. It inhibited the proliferation of HepG2, SSMC-7721, SK-HEP-1, and Bel-7402 cells, with a half-maximal inhibitory concentration (IC_50_) of 55.4, 48.9, 55.3, and 58.8 μg/kg, respectively [14].

A study investigated the apoptotic effects of the ethanol extract of *D. chinensis* (EEDC) in the human hepatocellular carcinoma cells HepG2 and found that treatment with 50, 100, 200, and 400 µg/mL EEDC for 6, 12, and 48 h induced time-dependent apoptosis. This induction was associated with the condensation of chromatin, activation of caspases, and cleavage of poly (ADP-ribose) polymerase protein. Moreover, apoptosis induced by *D. chinensis* was attenuated by a caspase inhibitor, indicating an important role of caspases in the effects of the methanol extract of *D. chinensis* (MEDC). Furthermore, *D. chinensis* did not alter the expression of B-cell lymphoma 2 (Bcl-2)-associated X protein (Bax) in HepG2 cells, but it selectively downregulated the expression of Bcl-2 and B-cell lymphoma-extra-large (Bcl-xl), resulting in an increase in the Bax:Bcl-2 and Bax:Bcl-xl ratios. These results support a mechanism in which *D. chinensis* induces apoptosis through the mitochondrial pathway and caspase activation in HepG2 cells [56].

The apoptotic activities and molecular mechanisms of action of MEDC were evaluated in human oral cancer cells using the 3-(4,5-dimethylthiazol-2-yl)-5-(3-carboxymethoxyphenyl)-2-(4-sulfophenyl)-2H-tetrazolium (MTS) assay, 4′,6-diamidino-2-phenylindole (DAPI) staining, immunostaining, western blotting, and reverse-transcription polymerase chain reaction (RT-PCR). Sp1 was significantly over-expressed in oral tumor tissues than in normal oral mucosa. The downregulation of Sp1 inhibited the growth of SCC-15 and YD-15 oral cancer cells, whereas *D. chinensis* inhibited cell growth and induced apoptosis in both cell lines by decreasing the expression of Sp1. In addition, treatment of the cells with MEDC (40 and 60 μg/mL) decreased Mcl-1 expression, which is a downstream target of Sp1, indicating that *D. chinensis* contains natural bioactive products that induce the apoptosis of tumor cells overexpressing Sp1 [57].

Li et al. analyzed the petroleum ether extract of *D. superbus* by MTT colorimetry and isolated eight components, which were found to have antitumor activity at 100 mg/L against HeLa, Smmc-7721, HepG2, SK-hep1, A549, and Bel-7402 cell lines. The fractions with a strong antitumor activity were analyzed using GC–MS, and their main chemical components were fatty acid esterification derivatives and phenolic compounds. These findings provide a scientific basis for the clinical use of Dianthi herba in the treatment of tumor diseases [44].

In conclusion, extracts of the effective parts of Dianthi herba have antitumor biological activity, which is of importance in explaining the material basis of the antitumor efficacy of Dianthi and provides evidence for the clinical use of *Dianthus* spp. in the treatment of tumor. However, it is necessary to strengthen research on the active ingredients and anticancer activity of Dianthi in vivo to provide a theoretical basis for the development and application of Dianthi.

### Antioxidant activity

Yun et al. studied the effect of *Dunhuang Yifang Qumai* decoction (20, 10, and 5 g/kg, respectively) on the nuclear factor erythroid-2 related factor 2 (Nrf2)/antioxidant response element (ARE) signaling pathway in a rat renal calcium oxalate stone model, dosed by gavage with 1% ethylene glycol and 2% ammonium chloride solution [58]. Superoxide dismutase activity, malondialdehyde level, and total antioxidant capacity in rat serum were evaluated using hematoxylin and eosin staining. The pathomorphological changes in the kidney tissue of rats in each group were examined. In addition, the kidney tissue expression of *Nrf2* and NAD(P)H:quinone oxidoreductase 1 (*NQO1*) mRNA was determined using RT-PCR; the protein expression of Nrf2 and ARE evaluated using immunohistochemistry. The results showed oxidative stress injury in the treatment groups that were administered high-, medium-, and low-doses of the Dunhuang medical prescription *Qumai* decoction, and its improving effect was dose-dependent [58]. The mechanism of the therapeutic action of *Qumai* decoction may involve the inhibition of kidney stone formation by upregulating the expression of factors related to the Nrf2/ARE signaling pathway, which provides a basis for its clinical use of *Qumai* in the prevention and treatment of kidney stones.

Flavonoids extracted from *D. superbus* using solid fermentation of *Aspergillus niger* were evaluated for antioxidant properties in vitro using the hydroxyl radical scavenging assay. The results showed that the scavenging ability increased in a concentration-dependent manner [59]. The IC_50_ value of the flavonoid against the hydroxyl radical was statistically determined to be 0.065 g/L. At concentrations of 0.05–0.20 g/L, the free radical scavenging ability of the buckwheat flavonoid increased in a concentration-dependent manner. The IC_50_ value for free radical scavenging was 0.046 g/L, indicating that *D. superbus* has a strong antioxidant activity [60].

In addition, Chen et al. used a Wistar rat model to examine the effect of *Qumai* decoction on lipid peroxidation in liver homogenates [82]. At a concentration of 3.13 mg/mL, the decoction had obvious inhibitory effects on lipid peroxidation in rat liver homogenates (*P* < 0.001) [61]. The antioxidant activity of components of different solvent fractions of the extract of *D. superbus* was studied, and each component exhibited varying inhibitory effects on free radicals. Components of the ethyl acetate (EtOAc) fractions exhibited a more potent effect than those of other fractions, and the IC_50_ indicated a strong antioxidant activity at 1.25 mg/mL [97, 98]. Furthermore, the scavenging effect of all components of the EtOAc fraction on hydroxyl radicals was significant [62].

The antioxidant activity of *Dianthus* is exerted through different mechanisms by scavenging free radicals and by upregulating factors related to the Nrf2/ARE signaling pathway; these findings provide a preliminary basis for the development of new natural antioxidants and the further development of anti-aging drugs [63, 64]. Therefore, although the antioxidant activity of *D. superbus* has been widely studied, it is necessary to further study the mechanism through which it reduces reactive oxygen species (ROS) and free radical formation, to provide a detailed theoretical basis for the practical application of *D. superbus*.

### Antiviral activity

Nile et al. studied the antiviral and cytotoxic activities of quercetin 3-glucoside (Q3G) isolated from *D. superbus* against influenza virus infection [22]. To determine the mechanism underlying the antiviral effect of Q3G on the influenza virus, time-dependent antiviral tests, molecular docking studies, virus-induced symptom analysis, pre-incubation, and screening of related gene expression were conducted [99]. Q3G from *D. superbus* showed strong antiviral activity against influenza A and B viruses. The IC_50_ value against A/PR/8/34, A/Victoria/3/75, A/WS/33, B/Maryland/1/59, and B/Lee/40 was 4.93, 6.43, 9.94, 8.3, and 7.1 μg/mL, respectively. It inhibited virus-induced cellular ROS generation and acidic vesicular organelles formation. Moreover, Q3G and oseltamivir, administered to the control group, were found to be cytotoxic [22]. The half-maximal cytotoxic concentration (CC_50_) value was > 100 μg/mL and nontoxic. In addition, Q3G did not inhibit the neuraminidase (NA) activity of the influenza virus but blocked viral replication, showing a more competitive binding affinity (− 8.0 kcal/mol) than guanosine triphosphate (GTP) (− 7.0 kcal/mol) in blocking the Pb2 subunit of influenza viral polymerase. Therefore, Q3G has a positive protective effect on infected host cells, and a strong inhibitory effect on influenza A and B viruses, which provides a new research direction on Q3G in the development of anti-influenza drugs.

Quercetin-7-*O*-glucoside (Q7G), a compound isolated from *D. superbus*, was analyzed for inhibitory effects on viral RNA replication using quantitative RT-PCR. The blocking effect of Q7G on the basic protein subunits of the RNA polymerase of influenza virus was detected using the AutoDock Vina program and M7GTP using a computer molecular docking assay. Q7G showed no cytotoxicity in MDCK cells (CC_50_ > 100 μg/mL), and its IC_50_ value was 3.1, 6.61, 8.19, and 5.17 μg/mL against influenza A/PR/8/34, A/Vic/3/75, B/Lee/40, and B/Maryland/1/59 virus strains, respectively. Treatment with Q7G highly reduced ROS and autophagy formation induced by influenza virus infection. Q7G does not bind directly to virus particles or affect NA activity. These results indicate that Q7G inhibits RNA polymerase and occupies the binding site of M7GTP on Pb2 [27]. In conclusion, *D. superbus* has strong antiviral activity as a TCM, especially the active compounds Q3G and Q7G. These findings provide a direction for further research on *D. superbus* as an antiviral drug.

### Anti-inflammatory activity

Shin et al. evaluated the anti-inflammatory effects of *D. superbus* fructus ethanolic extract (DSE) (200 mg/kg) on Th2-type cytokines, eosinophil infiltration, and other factors in an ovalbumin (OVA)-induced, murine asthma model [65]. To elucidate the potential mechanism of the anti-inflammatory effect of DSE, the expression of inducible nitric oxide synthase (iNOS) in the respiratory tract was also analyzed. DSE significantly reduced the levels of interleukin (IL)-4, IL-13, eotaxin, and immunoglobulin E (IgE); the number of inflammatory cells in the bronchoalveolar lavage fluid (BALF); level of inflammatory cell infiltration; and mucus production in the respiratory tract [100]. The results indicate that DSE also attenuates the overexpression of iNOS induced by OVA challenge. This suggests that DSE effectively protects against allergic airway inflammation by downregulating iNOS expression, and it has potential as a therapeutic agent for allergic asthma [65, 66].

In another study, to discover novel immunosuppressants, cytokine enzyme-linked immunosorbent spot and enzyme-linked immunosorbent assays were used to verify the dichloromethane soluble component of *D. superbus* (50 μg/mL) for suppressive effects against human alloreactive T cells [101]. The addition of *D. superbus* extracts to human mixed lymphocyte cultures caused a dose-dependent inhibition of proliferation and interferon *c* (IFNc) production by memory alloreactive T cells and increased the proportion of forkhead box P3-positive (Foxp3^+^) CD4^+^ T cells. To determine whether the extracts of *D. superbus* induce regulatory T cells, anti CD3/CD28-stimulated CD4 T cells were treated, and a dose-dependent upregulation of Foxp3 was found to be associated with novel suppressive effects. Mechanistically, extracts of *D. superbus* did not induce T cell IL-10 or transforming growth factor beta (TGFβ), but they blocked T cell protein kinase B (Akt) phosphorylation, a key signaling nexus required for T cell proliferation and expansion that simultaneously prevents *Foxp3* transcription. This study provides novel insights into the anti-inflammatory effects of *D. superbus* [67].

Slotkin et al. reported that lipophilic organic acids (20 μg/mL) and intermediate lipophilic organic acids (20 μg/mL) in aqueous extracts of *D. superbus* were highly effective in reducing IgE secretion by a human B cell line. Furthermore, preliminary in vivo studies showed that *D. superbus* reduced the symptoms of anaphylactic shock in peanut-sensitized mice [68].

Although basic animal studies are still needed to investigate the anti-inflammatory effect of *D. superbus*, its anti-inflammatory effect and potential action mechanism provide a basis for its potential application in the development of new drugs. In addition, its anti-inflammatory activity can be enhanced when it is used in combination with other drugs as a combined supplement [69].

### Diuretic activity

In selected hospitals, from 2007 to 2014, Xu et al. treated 200 patients with ureteral calculi who were allocated to the control and treatment groups using the random number table method [70], with 100 patients in each group. The patients in the control group were administered extracorporeal shock wave lithotripsy combined with rehydration, spasmolysis, and diuretic therapy. The results suggested that *D. superbus* decoction combined with extracorporeal shock wave lithotripsy for the treatment of ureteral calculi required a short treatment time and that the patients exhibited a low recurrence rate. This finding suggests that this treatment may be suitable for widespread clinical application [70]. Li et al. gavaged rabbits with *Qumai* decoction (2 g/kg), and after 60 min, the urinary output increased significantly, demonstrating an improved diuretic effect [71]. Deng et al. prepared a compound decoction containing *D. superbus*, and the effect was investigated using laboratory examinations such as routine urine analysis as well as clinical symptom evaluation. The results showed that the urinary tract symptoms were relieved following treatment with the decoction [72]. Other studies have proven that prescription drugs containing *D. superbus* exhibit diuretic effects that improve urinary tract symptoms [71, 73, 74].

### Uterine excitatory activity

Li et al. used a mouse model of early gestation to evaluate the effects of various doses of fruit extracts of *D. superbus* on embryo number and growth [75]. Mice in the gestation period were intragastrically administered various doses (8.5, 17, and 34 g/kg) of the extract once a day, and fruit extracts of *D. superbus* showed obvious effects on early pregnancy in mice. The fruit extract of *D. superbus* at the three doses increased the abortion rate in a dose-dependent manner. The anti-pregnancy effects of the fruit may be mediated by a reduction in pregnancy ketone levels, which prevents normal development of the decidua [75, 76]. Wang et al. acquired methyl 3,4-dihydroxybenzoate from *D. superbus*, using an in vivo mouse (pregnancy) uterus spontaneous life record as a model [38]. The result indicated that 200 μg/kg methyl 3,4-dihydroxybenzoate excited the pregnant uterus, which was monitored by uterine smooth muscle contraction strength and amplitude [38]. Guo et al. used in vitro rat and in vivo rabbit uterus models to investigate the synergistic effects of the extracts and prostaglandin E2 [77]. The experimental results indicated that alcohol extracts excited uterine smooth muscles more when combined with the drug, and the degree of contraction in the treatment group (4.33 ± 2.19 cm) was significantly higher than that before administration. Therefore, the ethanol extract of *D. superbus* has an obvious excitatory effect on the uterus of anesthetized free in vivo rats and the isolated peroneal muscle strips of rats. These studies show that Dianthi herba can stimulate the uterus, which is consistent with its traditional use in China to treat amenorrhea.

### Antimicrobial activity

The antimicrobial activity of various Chinese herbs against *Chlamydia trachomatis* in the urogenital tract was tested in a previous study, and the results showed that *Qumai* decoction exhibits high sensitivity and a minimum inhibitory concentration (MIC) of < 2 mg/mL. Furthermore, the volume and quantity of *Chlamydia* inclusions decreased gradually with increasing *Qumai* decoction concentration, and finally disappeared. *Chlamydia trachomatis* growth was found in the blank control pores, but not in the negative control wells [78]. Antimicrobial experiments with several strains were used to compare the plant-based cyclopolypeptide (XIII) from *D. superbus* with the standard drug, griseofulvin. Cyclopolypeptide (XIII) exhibited potent activity against the pathogenic fungus, *Candida albicans*, with an MIC of 6 µg/mL [79]. Moreover, the inhibitory effects of ethanol and water extracts of *D. superbus* on the activities of *Shigella dysenteriae*, *Bacillus cereus*, and *Vibrio cholerae* were studied. The MIC was 6.25–12.550 mg/mL, and the minimum bactericidal concentration (MBC) was 12.525–12.550 mg/mL. The results showed that the ethanol and water extracts of *D. superbus* had positive inhibitory effects on *S. dysenteriae*, *B. cereus*, and *V. cholerae* [10].

These studies show that Dianthi herba has antibacterial effects, but most of these studies have been carried out in vitro, and it is necessary to use modern technology to study the potential mechanism of action and to translate the in vitro and in vivo research findings into clinical applications.

### Other effects

Yun et al. isolated two bioactive compounds from *D. superbus*, 4-hydroxy-benzeneacetic acid and 4-methoxybenzeneacetic acid, to determine the components mediating its neuroprotective activity against glutamate-induced death of hippocampal neuronal HT22 cells. The two compounds effectively protected HT22 cells against glutamate toxicity [42]. Glutamate is obviously toxic to hippocampal neurons and was used to induce damage in the model used to determine the effects of *D. superbus* on cell viability*.* The cell viability was evaluated using the MTT assay. The EtOAc soluble fraction of *D. superbus* extract was obviously active, and it increased cell viability to 73.89% and 94.35% at concentrations of 10 and 100 µg/mL, respectively. These phenomena suggest that Dianthi herba may have neuroprotective activity.

Dianthi herba possesses several other biological activities such as hemolytic, anthelmintic, and antiallergic effects. In a previous study, a high concentration (100%) of *D. superbus* alcoholic extract showed slight hemolytic effects and insect-repellent activity at a dose of 2 mg/mL [80]. Yoon et al. studied the protective effect of EtOAc extract of *D. superbus* in renal inflammation and fibrosis; the extract at a dose of 10 or 50 mg/kg/day administered to *db*/*db* mice for 8 weeks significantly improved the blood glucose and insulin levels, insulin resistance, homeostasis model assessment index, and HbA1c level. Therefore, the extract can be used as a potential drug to treat glomerulonephritis and glomerulosclerosis, which lead to diabetic nephropathy [81]. Zhang et al. found that *Gualou Qumai* decoction reduced the level of C-reactive protein (CRP), IL-6, and TGFβ1 in the renal tissue of rats and exerted the therapeutic effect of alleviating inflammatory reaction and renal fibrosis. In a previous study, Dianthi herba combined with *Magnolia officinalis* cortex, *Aurantii fructus immaturus*, and other medicines was used to treat pancreatic hydatoncus diseases; the results showed considerable clinical curative effects of the combination [1]. The extract of *D. chinensis* (500 μg/mL) inhibited the production of IgE in vitro and in vivo in peanut-allergic mice, considerably reduced the allergic reaction caused by peanuts, and exerted a certain antiallergic effect [83].

In general, Dianthi herba has various pharmacological activities; however, screening and evaluation of its active compounds remain at the crude extract level. Research on its chemical composition, biological activity, pharmacodynamics, and mechanism of action remain limited. These gaps should be addressed in the future.

## Toxicity

Currently, there are only a few reports on the toxicity of Dianthi herba. The suggested daily dose of Dianthi herba is 9–15 g, and pregnant women should use it cautiously, as documented in the Pharmacopoeia of the PRC (Pharmacopoeia Commission of PRC, 2020). A study on the antifertility and genotoxic effects of Dianthi herba decoction in pregnant mice has shown that doses of 10, 15, and 30 g/kg considerably affected the implantation period and early pregnancy. Furthermore, 15 and 30 g/kg Dianthi herba exerted certain mid-term pregnancy-terminating effect, which was apparent in mice in the late pregnancy stage, where it shortened the gestation period and reduced the weight of offspring. This is consistent with the “abortion and inferior occlusive blood” effects of Dianthi herba recorded in Shennong's Classic Materia Medica. Dianthi herba caused abortion in mice in the early pregnancy stage, indicating the lethal effect of this drug [84, 102]. In addition, Dianthi herba can cause damage to the kidney. Previously, different doses (0.25, 0.5, and 2.5 g/mL) were used to determine the serum biochemistry of liver and kidney function. Organ index analysis and histopathology were performed to detect the morphological changes of the main organs in mice. qPCR was used to determine the role of organic anion transporters OAT1 and OAT3 in the toxic injury in mice treated with Dianthi herba. The results showed that the middle and high dose groups of Dianthi herba had different degrees of damage of the main organs in mice, especially the kidney was severely damaged [103, 104].

## Clinical applications

Clinical and human studies have shown that Dianthi herba is mainly used in the treatment of urinary diseases, diabetic nephropathy, edema, cysts, esophageal tumors, and rectal cancer. In piglets and lamb with dysentery, Dianthi herba has a unique curative effect. In a recent study on chronic prostatitis, 60 patients with damp-heat, stasis type, chronic prostatitis were selected and randomly divided into two groups, with 30 patients in each group. The mean age of the control group was 31.1 years and the mean course of the disease was 33 months. A *Gualou Qumai* pill and *Taohe Chengqi* decoction were added and removed for 8 weeks, at one dose a day, and 400 mL of extract was extracted by water decoction and administered twice, in the morning and evening, after a meal. The results showed that there are 27 columns (90%) were effective in the 30-column *Gualou Qumai* pill treatment group, indicating that the *Gualou Qumai* pill and *Taohe Chengqi* decoction can alleviate the symptoms of chronic prostatitis [3].

In a previous study, 60 patients with diabetic nephropathy, aged 40–75 years, with a duration of diabetes ranging from 4 to 10 years, were treated with a TCM decoction of modified *Gualou Qumai* (decocted in water twice) for 1 month, at one dose a day. Three hundred milliliters of warm decoction was administered twice, in the morning and evening. The results showed that there are 25 columns (83.33%) were effective among the 30 columns of treatment groups, indicating that the Jiawei *Gualou Qumai* decoction can effectively improve the clinical symptoms of patients and assist in alleviating albuminuria and renal mesangial lesions, and delaying the process of diabetic nephropathy.

**Fig. 13:**
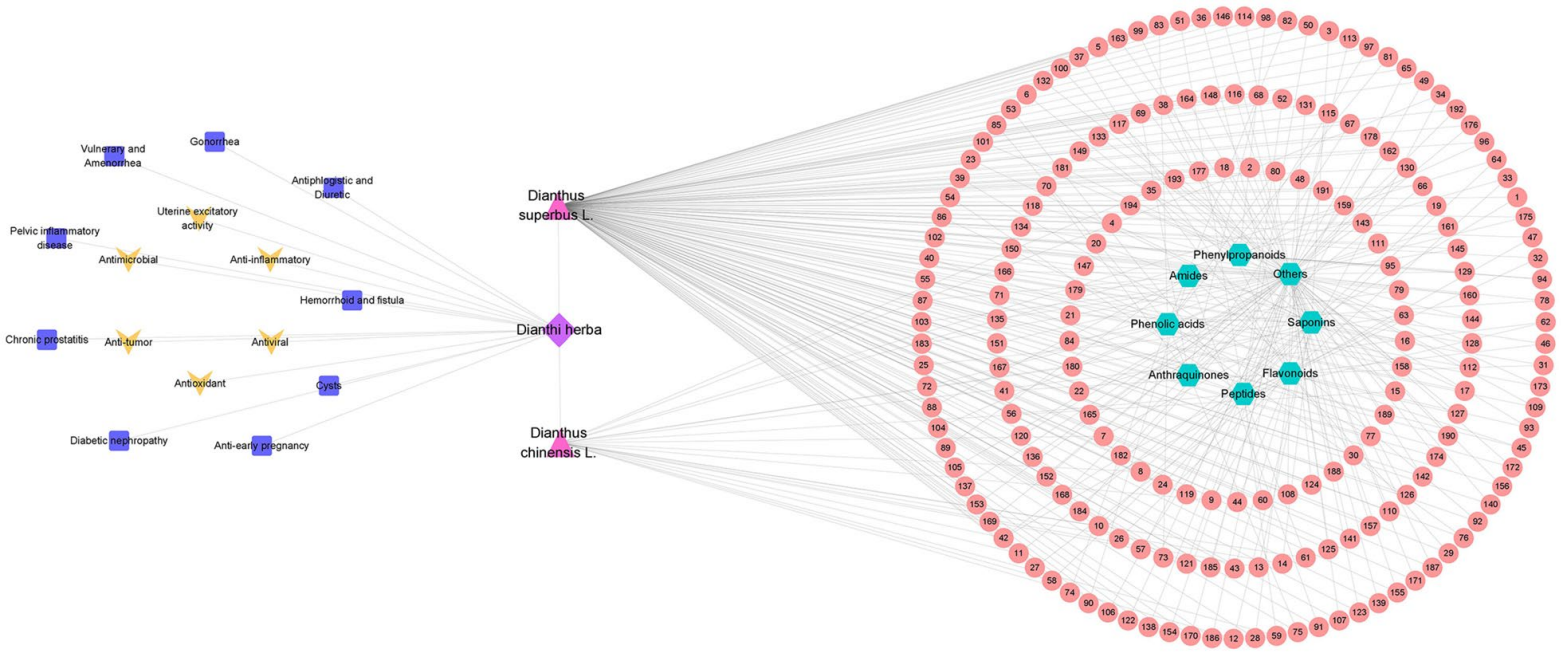
Sources, chemical constituents, pharmacological effects and traditional applications of Dianthi herba

Dianthi herba is also effective in the treatment of urinary retention after an anorectal operation [85]. Recently, 60 patients with urinary retention after anorectal disease were selected and were administered Qin Yanning granules (20 g each time). The patients’ first micturition time and voiding volume, *inter alia*, were measured. There are 55 (91.7%) patients was effective Among the 60 patients, indicating that Qin Yanning granules could significantly improve the micturition and abdominal distension symptoms of patients and improve the clinical effect [86]. Shoulin used *Gualou Qumai* pill to treat patients with edema, and the reexamination showed that the total protein and albumin level, 24-h urine volume, and liver function were substantially improved, indicating that the *Gualou Qumai* pill can effectively improve the clinical symptoms of the patients [87]. The network diagram of chemical composition, pharmacological action and traditional application of Dianthi herba is shown in Fig. 13.

## Conclusion and perspective

The traditional use, chemical composition, and extensive pharmacological activities of Dianthi herba are summarized based on information obtained from traditional literature records and modern literature. Dianthi herba has a long history of medicinal use in China. After decades of modern research, the main components such as saponins, flavonoids, peptides, anthraquinones and volatile oil have been isolated and identified, and their pharmacological activities have also been verified. Furthermore, through pharmacological experiments, the traditional applications of Dianthi herba have been confirmed, including the treatment of urinary tract infection and dysmenorrhea. However, more in-depth studies on the complex pharmacological effects of the *Dianthus* spp. and a complete phytochemical overview are needed to clarify the mechanism of action of Dianthi herba.

In modern times, *D. superbus* var. *longicalycinus*, *D. versicolor*, *D. amtifolia*, *D. chinensis* var. *liaotungensis*, and *D. shandongensis* are used as main sources of various medicines that can be confused with each other, and this could seriously affect treatment outcomes. According to the Chinese Pharmacopoeia, the identification methods for Dianthi herba are limited to thin layer chromatography and microscopy techniques. Therefore, it is necessary to establish an efficient, accurate, and scientific identification method to ensure the authenticity of the product. In addition, Dianthi herba medicinal materials are mostly found in wild medicinal materials. To ensure the conservation of sources of Dianthi herba medicinal materials and reduce the confusion of products and substitutes, attention should be paid to develop large-scale cultivation techniques. Studies should also focus on the establishment of a unified standard system and quality grade standards.

Saponins and cyclic peptides are considered the main pharmacologically active components in several bioactive compounds identified in Dianthi herba, and also new compounds isolated from Dianthi herba. Basic research on the pharmacological activities of Dianthi herba is limited, focusing mainly on the activity of the extracted parts. Therefore, the study on the biological activity of other chemical components and the interaction and structure–activity relationship between saponins and cyclic peptides should be strengthened in the future. In addition, clinical studies should be conducted to effectively evaluate the efficacy, adverse reactions, and toxicity of Dianthi herba.

Currently, Dianthi herba is mainly composed of aboveground parts, and little is known about the composition and activity of the underground parts. Therefore, in-depth studies on other parts of the plant should be carried out in order to reveal its overall development potential. Finally, in addition to high efficacy and less adverse effects, a qualified drug should have good pharmacokinetic properties, but there are only a few studies on the pharmacokinetics of Dianthi herba, which greatly limits the development of the plant in the future market of traditional Chinese medicine.

Herein, we summarized the traditional applications, chemical composition, and pharmacological action of Dianthi herba globally. Further exploration is still needed to guarantee the rational utilization of Dianthi herba. Moreover, considerable attention should be paid to market demand and quality control.

## Supplementary Information

**Additional file 1.** Distribution information of Dianthi herba.

## Data Availability

All data generated or analysed during this study are included in this published article [and its additional files].

## Abbreviations

TCM: Traditional Chinese Medicine
PRC: People’s Republic of China
CNKI: China National Knowledge Infrastructure
GBIF: Global Biodiversity Information Facility
GC–MS: Gas chromatography–mass spectrometry
NMR: Nuclear magnetic resonance
MTT: (3-(4,5-Dimethyl-2-Thiazolyl)-2,5-Diphenyl Tetrazolium Bromide)
MTS: 3-(4,5-Dimethylthiazol-2-yl)-5-(3-carboxymethoxyphenyl)-2-(4-sulfophenyl)-2H-tetrazolium
DAPI: 4’,6-Diamidino-2-phenylindole
RT-PCR: Reverse-Transcription polymerase chain reaction
Nrf2: Nuclear factor erythroid-2 related factor 2
ARE: Antioxidant response element
NQO1: NAD(P)H: quinone oxidoreductase 1
EtOAc: Ethyl acetate
ROS: Reactive oxygen species
Q3G: Quercetin 3-glucoside
CC_50_: The half-maximal cytotoxic concentration
NA: Neuraminidase
GTP: Guanosine triphosphate
Q7G: Quercetin-7-*O*-glucoside
OVA: Ovalbumin
iNOS: Inducible nitric oxide synthase
BALF: Bronchoalveolar lavage fluid
IFNc: Interferon c
TGFB: Transforming growth factor beta
MIC: Minimum inhibitory concentration
MBC: Minimum bactericidal concentration
CRP: C-reactive protein
